# Intra-Tumor Genetic Heterogeneity in Wilms Tumor: Clonal Evolution and Clinical Implications

**DOI:** 10.1016/j.ebiom.2016.05.029

**Published:** 2016-05-27

**Authors:** George D. Cresswell, John R. Apps, Tasnim Chagtai, Borbala Mifsud, Christopher C. Bentley, Mariana Maschietto, Sergey D. Popov, Mark E. Weeks, Øystein E. Olsen, Neil J. Sebire, Kathy Pritchard-Jones, Nicholas M. Luscombe, Richard D. Williams, William Mifsud

**Affiliations:** a The Francis Crick Institute, London, United Kingdom; b UCL Institute of Child Health, London, United Kingdom; c Department of Paediatric Haematology and Oncology, Great Ormond Street Hospital, London, United Kingdom; d UCL Genetics Institute, Department of Genetics, Evolution & Environment, University College London, United Kingdom; e Divisions of Molecular Pathology and Cancer Therapeutics, Institute of Cancer Research, London, United Kingdom; f Department of Radiology, Great Ormond Street Hospital, London, United Kingdom; g Department of Histopathology, Great Ormond Street Hospital, London, United Kingdom; h Okinawa Institute of Science & Technology, Okinawa, Japan

**Keywords:** Intra-tumor genetic heterogeneity, Pediatric solid tumors, Wilms tumor, Tumor evolution, Tumor multisampling, Molecular biomarkers, Copy number aberrations

## Abstract

The evolution of pediatric solid tumors is poorly understood. There is conflicting evidence of intra-tumor genetic homogeneity vs. heterogeneity (ITGH) in a small number of studies in pediatric solid tumors. A number of copy number aberrations (CNA) are proposed as prognostic biomarkers to stratify patients, for example 1q + in Wilms tumor (WT); current clinical trials use only one sample per tumor to profile this genetic biomarker. We multisampled 20 WT cases and assessed genome-wide allele-specific CNA and loss of heterozygosity, and inferred tumor evolution, using Illumina CytoSNP12v2.1 arrays, a custom analysis pipeline, and the MEDICC algorithm. We found remarkable diversity of ITGH and evolutionary trajectories in WT. 1q + is heterogeneous in the majority of tumors with this change, with variable evolutionary timing. We estimate that at least three samples per tumor are needed to detect > 95% of cases with 1q +. In contrast, somatic 11p15 LOH is uniformly an early event in WT development. We find evidence of two separate tumor origins in unilateral disease with divergent histology, and in bilateral WT. We also show subclonal changes related to differential response to chemotherapy. Rational trial design to include biomarkers in risk stratification requires tumor multisampling and reliable delineation of ITGH and tumor evolution.

## Highlights

• There is remarkable diversity of intratumor genetic heterogeneity and evolutionary trajectories.
• Gain of 1q is frequently heterogeneous and shows variable evolutionary timing.
• 11p15 CNNLOH is consistently an early event in Wilms tumorigenesis.
• Rational biomarker-based treatment stratification in Wilms tumors requires multisampling.
  We have shown that Wilms tumor (WT), the commonest pediatric kidney cancer, shows a range of evolutionary trajectories. We also found that gain of the long arm of chromosome 1 (1q +) may occur both early or late in the evolution of WT. 1q + is associated with a poorer outcome and is proposed for use in choosing patients for more intensive treatment, but we show that in order to detect it reliably, future trials require testing for this biomarker in multiple samples per tumor.

## 1 Introduction

Intra-tumor genetic heterogeneity (ITGH) has been documented in several adult tumors. Such tumors typically evolve over long periods before diagnosis, with most demonstrating branched evolutionary trajectories (Nowell, 1976, Greaves and Maley, 2012, Gerlinger et al., 2012, Gerlinger et al., 2014, de Bruin et al., 2014). However, the prevalence and relevance of ITGH are poorly understood in pediatric solid tumors: since they carry lower burdens of mutational changes and have evolved for shorter periods of time before diagnosis, they may be expected to show less complex evolutionary histories (Vogelstein et al., 2013).

Although there are relatively few sequence mutations in pediatric malignancies, DNA copy number aberrations (CNA) and rearrangements are often characteristic features of these tumors. Some common CNA have recognized prognostic significance in pediatric tumors. For example, in neuroblastoma, *MYCN* amplification or subchromosomal genomic gains and losses are used to stratify therapy (Schleiermacher et al., 2011). In Wilms tumor (WT), gain of 1q (1q +) is increasingly being proposed as a common prognostic biomarker to select patients for more intensive treatment (Natrajan et al., 2006, Gratias et al., 2013, Segers et al., 2013). However, these studies have relied on a single tumor sample from each case.

Indeed, there has been limited investigation of ITGH in pediatric solid tumors. A recent multisampling study reported genetic homogeneity in multi-sampled embryonal brain tumors (Remke et al., 2015). However, a study of four pediatric small round cell tumors, with two samples from each, reported heterogeneous CNA in three out of four tumors (Mengelbier et al., 2015).

In WT, a large study showed that combined loss of heterozygosity (LOH) of chromosomes 1p and 16q, while rare, was not only associated with poorer outcome, but also showed concordance in the vast majority of the 10% of tumors from which two separate samples were assessed (Grundy et al., 2005). In contrast, heterogeneous *WTX* deletion has been reported in two multi-sampled WTs (Wegert et al., 2009), and heterogeneous activation of *MYCN* and inactivation of *TP53* have been reported in a case of bilateral WT ( Popov et al., 2013, Williams et al., 2015).

Such variable heterogeneity complicates clinical decision making because of a poor understanding of the evolution of pediatric tumors. It also means that most previous studies showing prognostic significance for specific CNA did not take into account potentially significant ITGH. Therefore, here we assess the extent and significance of ITGH in a prospective study of unselected multi-sampled Wilms' tumors.

## 2 Methods

### 2.1 Samples

We obtained multiple samples from WT nephrectomy/nephron-sparing surgery specimens at Great Ormond Street Hospital between May 2011 and June 2013. All patients were enrolled on the SIOP WT 2001 trial (Pritchard-Jones et al., 2015), the current IMPORT study or their parents had consented for additional tissue to be used in research as part of the UK Children's Cancer and Leukaemia Group tissue bank. The research reported here was approved by a national research ethics committee. Patients received preoperative chemotherapy as per the SIOP WT 2001 trial protocol or according to national clinical guidelines based on this trial. Tumors were classified as previously described (Vujanić et al., 2002). A histological section from each tissue sample was reviewed to determine viable tumor content, and only samples with more than 50% viable tumor (the remainder consisting of necrotic tumor/post-chemotherapy change) were used. DNA was extracted using standard techniques from each tumor sample, and from adjacent non-tumorous kidney where it was available in 19 cases, and from peripheral blood lymphocytes in 3 cases.

### 2.2 Imaging

In two cases, different regions within the same tumor were identified prospectively as distinct nodules in the same overall tumor mass on T1- and T2-weighted MR imaging, and matched on comparison of pre- and post-chemotherapy images. Assessed diffusion coefficient (ADC) was calculated by one observer (ØEO) as previously described (McDonald et al., 2010).

### 2.3 Molecular Analyses

Illumina® HumanCytoSNP-12 v2·1 microarrays (~ 300,000 SNP probes) were hybridized with 250 ng DNA per sample according to the manufacturer's instructions. Methylation-specific multiplex ligation-dependent probe amplification (MS-MLPA) for 11p15 was carried out as previously described (Scott et al., 2008a), using the Salsa MS-MLPA BWS/RSS ME030-C3 probemix (MRC-Holland), and data visualized in Coffalyser.NET (MRC-Holland).

### 2.4 Detection of Allele-Specific Copy Number Aberrations and Inference of Tumor Evolution

Log R ratio (log_2_[observed intensity/reference intensity], LRR) and B-allele frequency (BAF) were calculated using the Illumina® GenomeStudio software for each array using default settings. LRR genomic waves (Diskin et al., 2008) were detected in normal tissue samples and corrected from all arrays. The LRRs from each array were segmented and copy number states were called using the ‘CGHcall’ R package (van de Wiel et al., 2007) in Bioconductor (Gentleman et al., 2004). For each case, the boundaries between adjacent regions were compared, smoothed and summarized between samples using the ‘CGHregions’ R package (van de Wiel and van Wieringen, 2007) in Bioconductor (Gentleman et al., 2004). A region was removed if it contained fewer than 100 probes or its probe density was an outlier. The mean tumor-specific mirrored BAF (mBAF) was calculated for each aberrant region and copy number aberrations were rejected if they did not show expected allelic imbalance. Aberrant regions detectable only in the BAF (i.e. copy number neutral LOH) were incorporated into our analysis using a custom pipeline. Allele-specific copy number was interpreted by the phylogenetic algorithm MEDICC (Schwarz et al., 2014) to infer clonal evolution of samples in each case. Normal tissue samples were used to root phylogenetic trees. Annotated code of our entire analysis pipeline is available as a GitHub repository at: https://github.com/luslab/multiregion-cnv-phylogenetics.

### 2.5 Role of the Funding Source

The study sponsors did not participate in study design, in the collection, analysis, and interpretation of data, in the writing of the report, or in the decision to submit the paper for publication.

## 3 Results

### 3.1 Intra-Tumor Genetic Heterogeneity in Wilms Tumor Demonstrates Unexpected Diversity

**Fig. 1:**
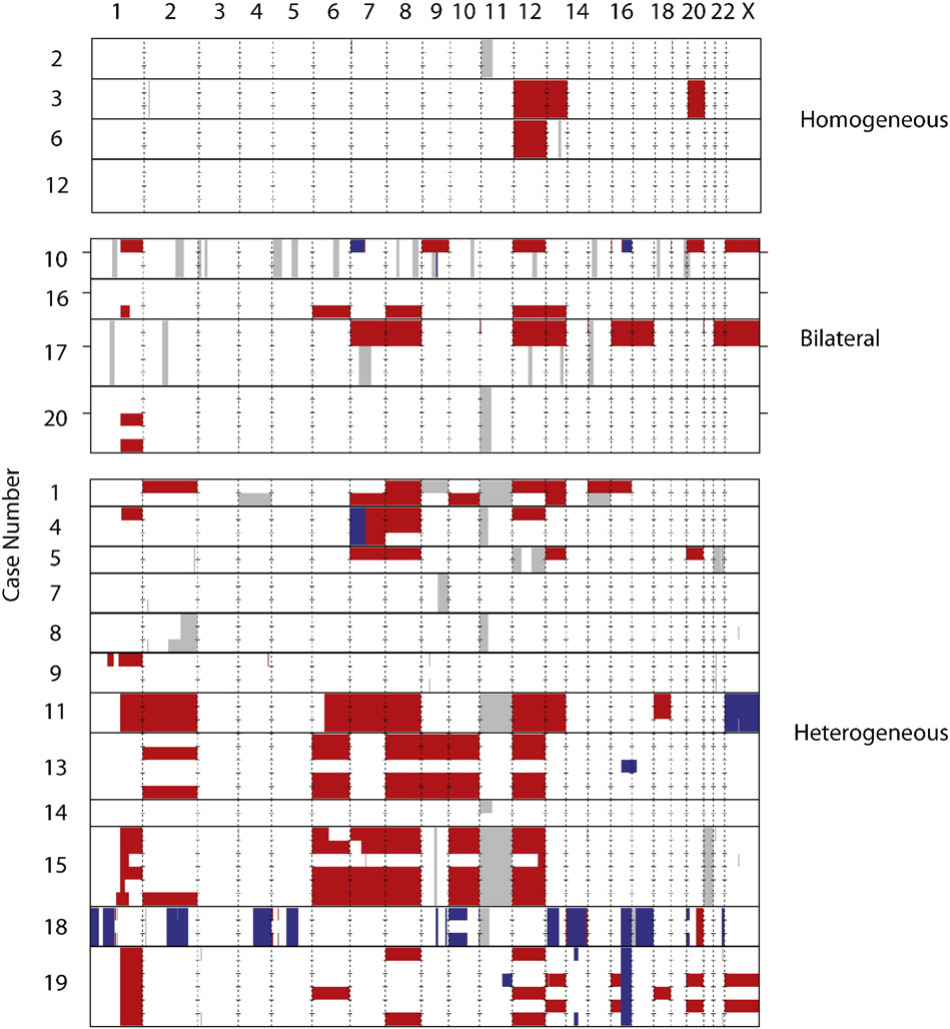
Scope of intra-tumor copy number heterogeneity in 20 Wilms tumor cases. Case numbers, indicated on the y-axis, are split into three groups i) homogeneous, ii) bilateral and iii) heterogeneous. Chromosomes 1 to 22 and X are ordered on the x-axis from left to right, separated by vertical dashed lines. Cases are separated by solid black horizontal lines and samples from each case by dashed horizontal marks at each chromosome boundary. In the bilateral group, samples from contralateral kidneys are split by horizontal markers either side of the plot. Copy number states are displayed as white (expected copy number state), red (gain state), blue (loss state) and grey (expected copy number state with loss of heterozygosity).

We studied 70 distinct tumor samples from 24 tumors in 20 patients (mean 3.5 samples/case, range 2–6 samples), with matched DNA from non-tumorous kidney and/or peripheral blood leukocytes in 19 cases. Five patients (Cases 9, 10, 16, 17, 20) had bilateral WT, and we obtained samples from both tumors in four of them; in Case 9, the contralateral tumor had been removed prior to the start of our study. Patient characteristics and samples are summarized in Table 1. We applied a custom-built pipeline to determine reproducibly genome-wide allele-specific CNA and LOH events using high-resolution SNP arrays hybridized with genomic DNA from each sample, and automatically compare these events across samples in a tumor. Fig. 1 shows a graphical representation of all CNA and copy number neutral LOH (CNNLOH) events across the 70 tumor samples. We detected most known recurrent WT CNA/LOH, including those associated with poor outcome (Natrajan et al., 2006, Gratias et al., 2013, Segers et al., 2013, Grundy et al., 2005). Surprisingly, 1q + was heterogeneous in four of seven (57%) multi-sampled tumors with this change (see below). In general, we found remarkable diversity in the extent of intra-tumor CNA and CNNLOH heterogeneity, ranging from cases with unique CNA/CNNLOH events in all or most samples (such as Cases 1 and 15) to tumors exhibiting no CNA/CNNLOH heterogeneity across samples. The latter either lacked somatic CNAs/CNNLOH (Case 12) or showed a consistent pattern of somatic CNAs/CNNLOH across all samples (Cases 2, 3, 6), and the single dominant clone in each tumor showed few (0–4) CNAs/CNNLOH events. The four patients with these homogeneous (and unilateral) tumors were not statistically significantly younger than the other twelve patients with heterogeneous unilateral tumors (Welch two-sample one-tailed *t*-test, *t* = − 1.52, p = 0.08), suggesting that heterogeneity does not arise purely as a consequence of later age at diagnosis.

### 3.2 Diverse Evolutionary Pathways in Wilms Tumor: From Homogeneity to Branched Evolution

In order to infer the evolutionary history of tumors, we generated phylogenetic trees depicting the relationships between samples. Our pipeline requires a minimum of four samples, and this condition was satisfied in seventeen cases. In thirteen cases, all tumor samples were from the same kidney (unilateral). We obtained a flat phylogenetic tree, where all samples contain the same clone, in Cases 3, 6 and 12. Cases 7 and 9 gave a linear tree, in which clones are derived from ‘ancestral’ clones sharing all prior evolutionary events. Branched evolution, in which more than one clone contains unique events compared with a common ancestor, was observed in the remaining seven unilateral tumors (1, 4, 8, 11, 13, 15, 18, 19).

**Fig. 2:**
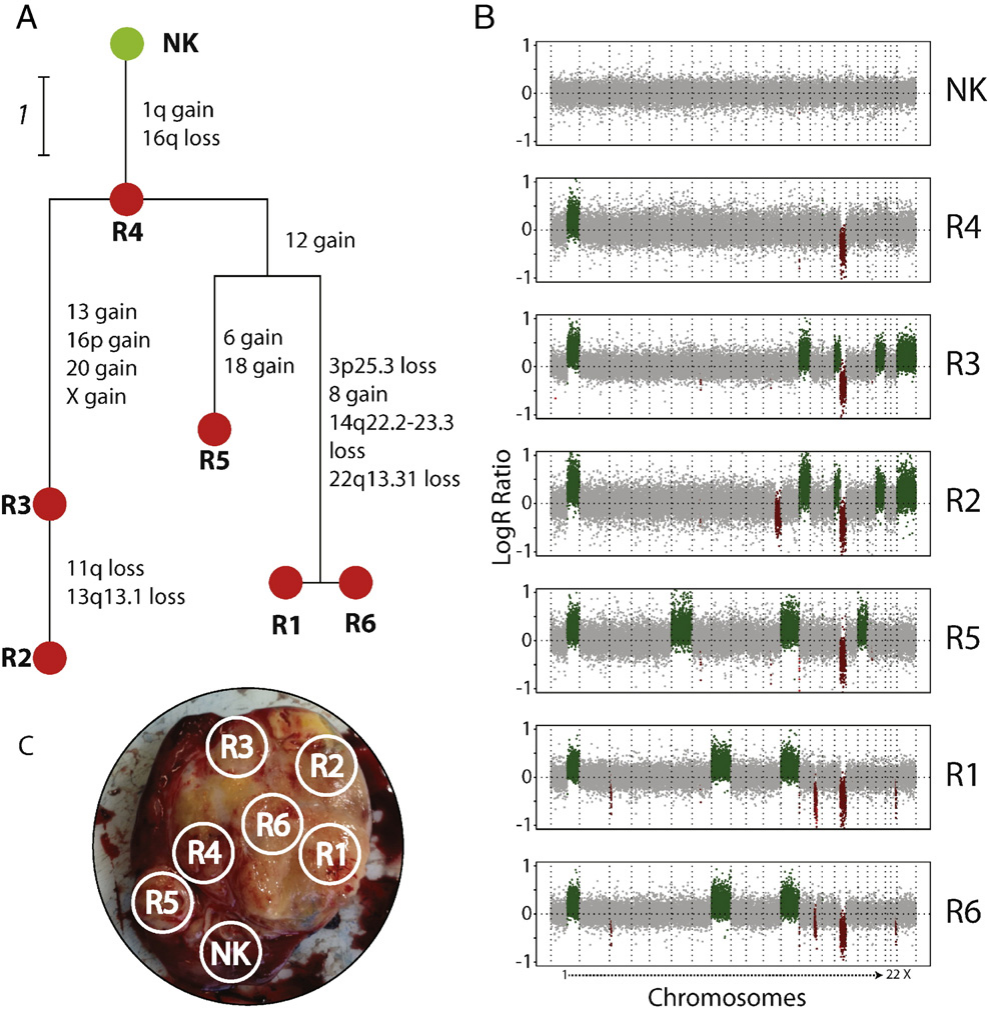
Branched evolution of a multi-sampled Wilms tumor from Case 19. (A) Phylogenetic tree displaying the evolutionary relationship between the normal kidney sample (NK, green node) and tumor samples (R1–6, red). Vertical edges are weighted by the number of copy number alterations that were acquired as the tumor evolved and the events themselves are labelled next to the appropriate edge. Horizontal edges are not weighted and are used to separate graphically the nodes. (B) Copy number profiles of the normal kidney and tumor samples. Plots display the Log R ratio on the y-axis and chromosomes 1–22 and X on the x-axis. Data are shown for every tenth SNP probe. Data points corresponding to normal diploid copy number states are coloured grey, those representing copy number gains are in green and losses in dark red. (C) An annotated photograph of the opened nephrectomy specimen, showing the locations of the sampled tumor and normal kidney regions.

Branched evolution is particularly well demonstrated by Cases 15 and 19, with marked branching that began early in the clonal evolution of the tumor. In Case 19 (Fig. 2), the most recent common ancestor clone observed in sample R4 shows 1q + and 16q −. All other tumor samples display extra CNAs: R3 and R2 share four additional events (+ 13, 16p +, + 20, + X) and cluster separately from R5, R1 and R6. R2 contains a clone with two additional events (11q − and 13q13.1 −) to R3. Interestingly, 13q13.1 loss removes a specific region encompassing *BRCA2*, after previous gain of the entire chromosome 13. R5, R1 and R6 share a + 12 event that explains the distinct branching of the samples. However, further branching is seen in these samples as R5 contains two unique events (+ 6, + 18), and R1 and R6 both exclusively share four events (+ 8, 3p25.3 −, 14q22.2–23.3 −, 22q13.31 −). The clone present in R1 and R6 acquired three focal losses late in its evolution that encompass known cancer genes such as *VHL*, a tumor suppressor gene commonly mutated in clear cell renal cell carcinoma (ccRCC) (Gnarra et al., 1994), and *SIX1*, a gene that was recently reported to be recurrently mutated in WT (Wegert et al., 2015, Walz et al., 2015) (3p25.3 − and 14q22.2–23.3 −, respectively). Case 15 is even more diversified, with unique events in five of six samples (Supplementary Fig. 1).

### 3.3 Genetic Markers and Evolutionary Timing

#### 3.3.1 Chromosome 11p15 Uniparental Disomy is Consistently an Early Event in WT Tumorigenesis

We observed 11p15 CNNLOH in 9 of 20 cases (Fig. 1). In six of these cases, LOH also involved the entire 11p13 region, and in Case 18, it involved part of the 11p13 region, including the *WT1* locus; in Cases 4 and 8, 11p LOH did not involve 11p13. In all but Case 14, 11p15 CNN LOH was detected in all tumor samples, indicating that it occurred in a common ancestor of all observed clones in these tumors, and is thus a consistent early event in WT tumorigenesis. The observation that 11p15 CNNLOH was a truncal event in the evolution of these WTs was confirmed by phylogenetic analysis. In Case 14, 11p15 CNNLOH was called in just one of the two samples taken from this tumor, where it was the only CNA observed (Fig. 1). Visual inspection of the chromosome 11 BAF plots for this case shows that despite not being identified in one of the samples by our computational analysis, the event does appear to be present in a low fraction of cells (Supplementary Fig. 2). Therefore, Case 14 does not contradict the evidence from other cases that 11p15 CNNLOH is a consistently early event in WT tumorigenesis.

Since chromosome 11p15 contains a cluster of imprinted genes, we performed MS-MLPA on the 11p15.2 locus (Scott et al., 2008a) in all cases with 11p CNNLOH, and in all tested samples found uniparental disomy (UPD), as indicated by hypermethylation of the *H19* DMR (consistent with overexpression of the *IGF2* oncogene) and hypomethylation of the KvDMR, (i.e. a paternal pattern). DNA from adjacent normal kidney was available for eight of the nine cases with 11p15 CNNLOH, and in all eight cases, there was neither 11p CNNLOH nor abnormal methylation, indicating that the CNNLOH and methylation abnormalities were somatic events in the tumor cells.

In contrast, MS-MLPA on the cases without somatic 11p15 CNNLOH showed five cases (Cases 6, 10, 13, 17, 19) with hypermethylation of the *H19* DMR only, with normal methylation of KvDMR. In these cases, the abnormal methylation pattern was also present in adjacent histologically normal kidney. Case 6 had known hemihypertrophy, but the other cases had not been diagnosed with Beckwith-Wiedemann syndrome, hemihypertrophy, abnormal growth, macroglossia, hypoglycemia or other tumors.

### 3.4 Gain of Chromosome 1q Shows Variable Timing

**Fig. 3:**
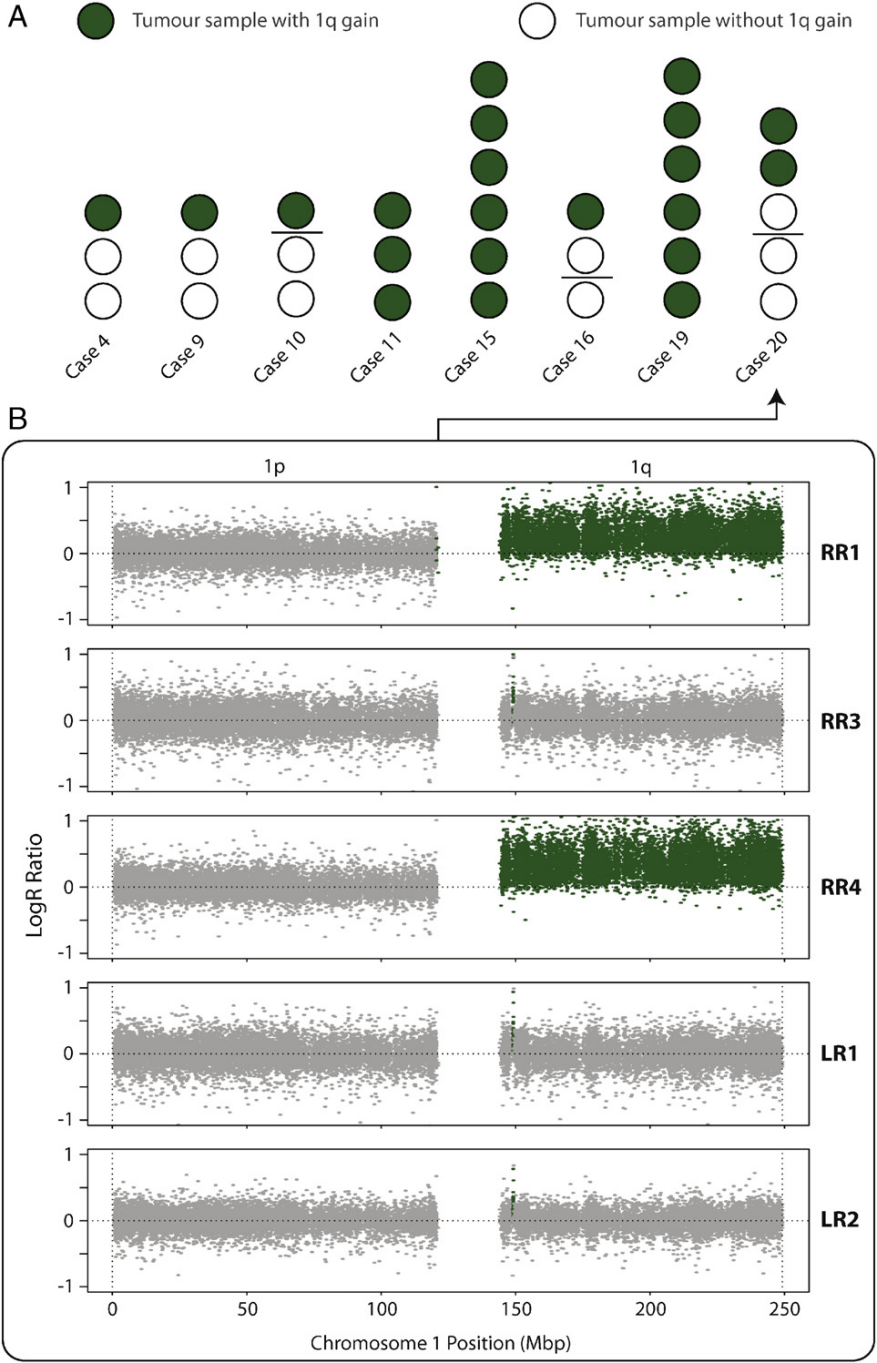
Chromosome 1q gain (1q +) is variably heterogeneous in eight cases. (A) Each sample is shown as a circle, filled in green if there is 1q + and in white if there is not. Bilateral cases are split into contralateral samples with a horizontal black line. (B) Chromosome 1 copy number profiles for samples from Case 20. Log R ratios (y-axis) are shown for all chromosome 1 SNP probes, ordered by genomic position on the x-axis. Data points are coloured green if they correspond to copy number gains, and grey if not. Samples R1 and R4 display whole chromosomal arm gains.

We observed 1q + in eight patients (40%, Fig. 3; Supplementary Fig. 3). For seven of these we had multisampled the tumors in which we detected the change. 1q + is present in all the tumor samples in three of these cases (Cases 11, 15, 19). In Cases 11 and 19 the same 1q + is in all samples. In Case 15 there is 1q + in all tumor samples (n = 6); however, there are several unique CNAs that affect this chromosome arm, thus displaying ITGH in the extent of 1q + itself. In the remaining four cases, 1q + is present in one of three (Cases 4 and 9), one of two (Case 16, right kidney tumor) and two of three (Case 20, right kidney tumor) samples. Thus, if we had single sampled our tumors, we would have only definitely identified three of seven tumors with 1q +; the probability of finding 1q + in all seven tumors with single sampling is only 0·037. Indeed, the average probability of obtaining a negative single sample per tumor in our set of seven multi-sampled tumors with 1q + is 0.31 (95% confidence interval is 0.08–0.54), despite 1q + being present in all samples obtained from three of the seven positive tumors. The cumulative binomial distribution with p = 0.69 indicates that at least three samples per tumor must be studied in order to ensure that greater than 95% of tumors with 1q + are detected.

In the four tumors which display 1q + ITGH, 1q + shows no preference in evolutionary timing. In Cases 9 and 16 chromosome 1q + timing is indistinguishable from other copy number changes present in the tumor because samples from these tumors represent either the only clone with tumor-specific CNAs or have a copy number profile that is indistinguishable from matched germline DNA. In Cases 4 and 20 1q + follows other CNAs (e.g. UPD of chromosome 11p in both cases) indicating that 1q + evolved late. In contrast, in Cases 11 and 19 1q + is a truncal event that may have occurred early in tumor evolution.

### 3.5 Rarer Biomarkers

Other WT biomarkers are difficult to assess without larger patient cohorts. Nevertheless, we observed 16q − in four tumors, three of which are multi-sampled (Fig. 1). Of these, Cases 18 and 19 show homogeneous 16q −, accompanied in Case 18 by homogeneous 1p − and 4q −. In Case 13, 16q − is apparently heterogeneous, but the change is present in the one sample that is from a tumor that probably originated independently in the same kidney (see below); therefore, we have no evidence to support 16q − as a heterogeneous event. Two cases contain a LOH event that affects the *TP53* locus: homogeneous 17p CNNLOH in Case 18 and apparently heterogeneous loss of *TP53* in Case 13 occurring in the single sample from the tumor with probable independent origin. Case 18 showed diffuse anaplasia on histological examination, whereas Case 13 showed neither diffuse nor focal anaplasia or nuclear unrest. Focal *MYCN* gain is heterogeneous in Cases 7 and 8 and homogeneous in Cases 3 and 16. *MYCN* gain is germline in Case 16 (previously reported (Williams et al., 2015)).

### 3.6 Spatially Separated Tumors

#### 3.6.1 Bilateral Tumors

Five cases in our dataset are bilateral WTs, and in four of these both sides were sampled (Cases 10, 16, 17 and 20). Overall, bilateral tumors appear genetically distinct and probably arose independently. Cases 10 and 17 provide the clearest examples of the striking differences in copy number profiles between contralateral tumors. In Case 10 the only tumor-specific CNAs in the two samples taken from the left tumor are two focal deletions in chromosome 9, whereas the sample from the right tumor shows 1q +, + 9, + 12, + 20, + X, 7p − and 16q −. In Case 17, the left tumor of Case 17 shows + 7, + 8, + 12, + 13, + 16, + 17, + 22 and + X, while the right tumor shows no tumor-specific CNAs. In both these cases, the two contralateral tumors share no tumor-specific CNAs and are not heterogeneous within the individual kidneys. Case 16 only showed CNAs in one sample of two taken from the right tumor, thus making the right tumor heterogeneous. The left tumor sample of Case 16 did not have any CNAs.

Case 20 was heterogeneous within the right tumor, with 11p CNNLOH in all three samples but 1q + in only two samples. The left tumor also contained 11p CNNLOH, but with a different centromeric boundary, indicating this may have been a separate unrelated event in the left tumor (Supplementary Fig. 2). However, we cannot formally exclude the possibility that the tumors in the two kidneys developed from a single clone with the shorter 11p CNNLOH, showing no further evolution in the left tumor and extension of 11p CNNLOH in the right kidney.

### 3.7 Evidence for Two Separate Tumors in the Same Mass

**Fig. 4:**
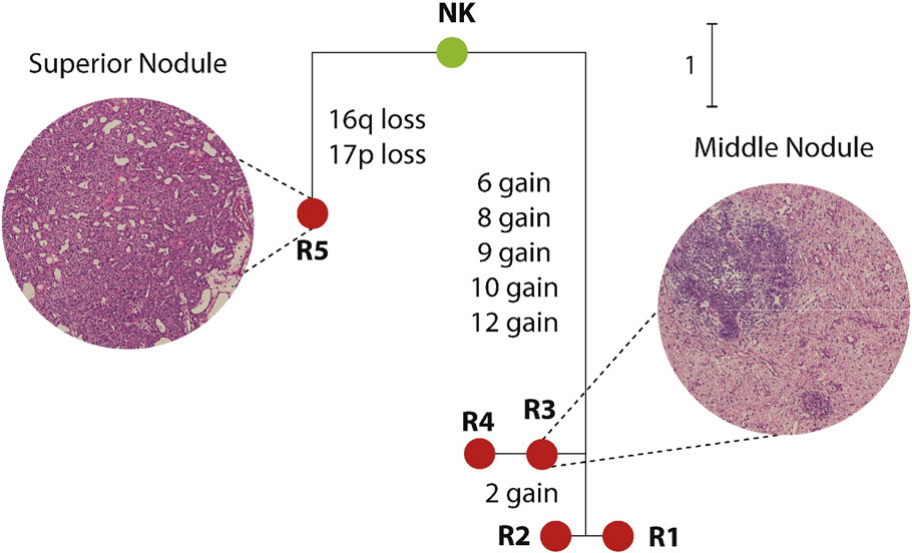
Phylogenetic analysis reveals two genetically distinct Wilms tumors within a single mass in Case 13. The phylogenetic tree shows the evolutionary relationship between the normal kidney sample (NK, green node) and tumor samples (R1–5, red). The edges were drawn using the same rules as in Fig. 2. The tree shows that sample R5 is genetically distinct from R1–4, since their only common ancestral state corresponds to the normal kidney sample. The histology of the middle nodule, represented by R3, is triphasic, with blastemal, epithelial and stromal elements, whereas the superior nodule, represented by R5, is markedly different and composed exclusively of more mature epithelial elements (original magnification, × 100).

Case 13 presented as one tumor mass consisting of a larger middle nodule, consisting of mixed blastemal, epithelial and stromal elements, contiguous with a superior nodule composed of relatively well-differentiated epithelial structures, thus showing two contrasting WT phenotypes in the same apparent tumor. Four samples were obtained from the middle nodule and one from the superior nodule. The samples from the middle nodule were heterogeneous due to the presence or absence of + 2, with homogeneous + 6, + 8, + 9, + 10, + 12. The sample from the superior nodule contained 16q − and 17p −. Thus, the two nodules showed independent branching from germline DNA, suggesting that they arose independently, despite appearing as one contiguous tumor (Fig. 4).

#### 3.7.1 Relation to Treatment Response

**Fig. 5:**
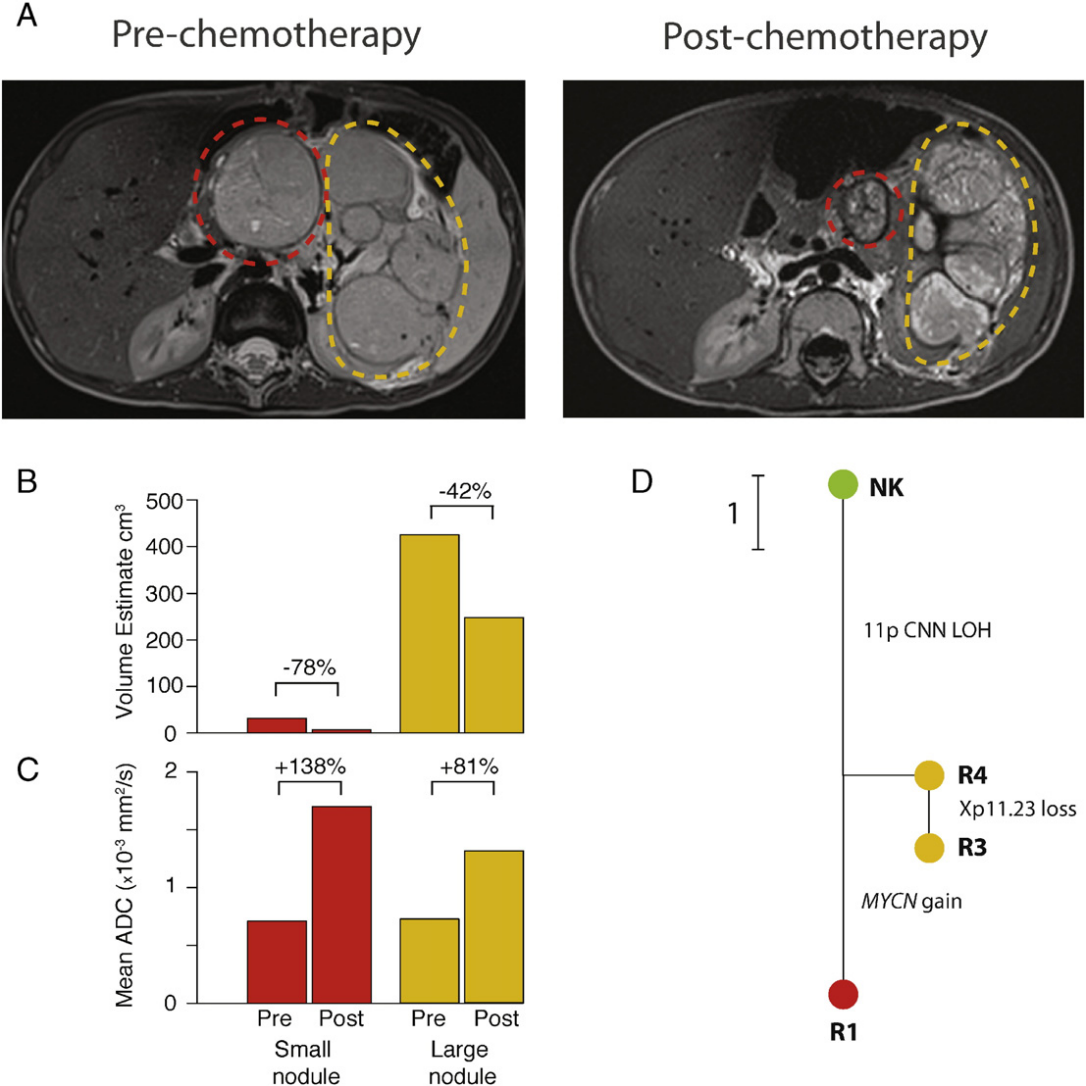
Wilms tumor phylogeny mirrors the differential treatment responses shown by different parts of the same tumor in Case 8. (A) Pre- and post-chemotherapy T2-weighted magnetic resonance (MR) images show the two masses indicated by red (small nodule) and orange (large nodule) dashed lines. (B) Estimated tumor volumes pre- and post-treatment shows that the small nodule (red) shrank by 78% and the large nodule shrank by 42% (orange). (C) The small nodule (red) showed a greater post-chemotherapy gain in the mean assessed diffusion coefficient (ADC) than the large nodule (orange), indicating a better response to chemotherapy. (D) A phylogenetic tree shows the genetic relationships between samples from the large nodule (R3–4, orange) and the small nodule (R1, red). The normal kidney sample is represented by a green node (NK). The small nodule is related to the rest of the tumor, but has evolved additional changes, including focal gain of *MYCN*.

Case 8 also presented with a large intrarenal mass contiguous with a smaller nodule (Fig. 5A). Following neoadjuvant chemotherapy, however, the smaller nodule showed a greater shrinkage (78% vs. 42%, Fig. 5B) and increased ADC (0.71 to 1.71 vs. 0.73 to 1.32, Fig. 5C) than the main tumor mass, indicating a better response to chemotherapy (McDonald et al., 2010, Hales et al., 2015). Histologically, all areas of the tumor showed a mixed histology.

We obtained one sample from the smaller nodule (R1) and two from the larger mass (R3, R4); they show ITGH and phylogenetically, R4 represents a clonal ancestor of the other two samples. R3, also from the larger mass, evolved an additional Xp11.23 −, while the smaller nodule developed 2q CNNLOH and a *MYCN* gain.

## 4 Discussion

Short-term outcomes in pediatric cancers are better than in adult cancers, but relapses are often unpredictable, and most current treatment protocols depend on cytotoxic agents with significant long-term complications (Reulen et al., 2010). There is a need to select better those patients who need more/less intensive therapy, and to increase the use of potentially safer targeted therapies. In WT, 1q + is a promising genetic biomarker for such stratification, because it is both common and associated with a poorer outcome. Nevertheless, to date, assessment of its prognostic significance and suggested use in clinical trials has assumed that single tumor sampling is representative and sufficient. There is emerging evidence from a small number of recent studies that in WT and other pediatric solid tumors, a subset of cases show ITGH. We therefore hypothesized that ITGH in WT may involve prognostically significant CNA such as 1q +, and therefore we prospectively multi-sampled a series of twenty WT cases in our center. In parallel, we developed a custom analysis pipeline to detect reliably CNA and LOH events, compare them across multiple samples for each tumor, and infer evolutionary trajectories in order to provide a basis for understanding how ITGH arises. We found a remarkable range of evolutionary scenarios and variable ITGH in WT, including 1q +. Indeed, our data indicated that single sampling misses a significant proportion of cases with 1q +, and we estimated that to detect more than 95% of cases with 1q +, one would need to obtain at least three tumor samples per case.

We also found that 1q + does not show preference in evolutionary timing—it may occur as an early or late event—which suggests that its oncogenic effect is independent of other genetic changes. Therefore, we suspect that 1q + may have a similar effect on WT outcome regardless of whether it is homogeneous or heterogeneous in the primary tumor, and that current studies based on single tumor sampling may have underestimated its prognostic significance. Our findings clearly imply that future clinical trials in WT must take this heterogeneity into account and multi-sample each tumor or attempt to detect this change in circulating tumor DNA at a level that can interrogate subclonality.

In contrast, we find that somatic 11p15 CNNLOH, another common change in WT, is consistently an early event in WT tumorigenesis. Our finding therefore builds on previous observations of somatic 11p CNNLOH in WT precursor lesions (nephrogenic rests) (Charles et al., 1998). 11p15 CNNLOH is associated with several other pediatric small round cell tumors, and recently it was found as a recurrent lesion in the vast majority of pediatric adrenocortical tumors, also occurring as an early event preceding most point mutations (Pinto et al., 2015). These findings suggest that 11p15 CNNLOH may represent a common mechanism of tumorigenesis in a significant proportion of pediatric solid tumors, and its occurrence as an early event makes it a promising candidate for early detection of pediatric cancer by non-invasive screening for ctDNA in blood.

In those tumors without 11p15 CNNLOH we identified a subset of five cases with isolated hypermethylation of the *H19* DMR, and this abnormality was also present in adjacent histologically normal kidney. In one of these five cases, there was hemihypertrophy, whereas in the other four cases there were no features to suggest Beckwith-Wiedemann syndrome. This finding is in keeping with previous reports of mosaic hypermethylation of the *H19* DMR in a significant proportion of normal cells in cases of WT with this abnormality, even in the absence of other features of Beckwith-Wiedemann syndrome (Moulton et al., 1994, Okamoto et al., 1997, Scott et al., 2008b). Indeed, the proportion and distribution of non-tumor cells with this WT-predisposing epimutation may at least in part underlie the expression and variability of the features of Beckwith-Wiedemann syndrome.

We have uncovered evidence of independent origins of two synchronous WT not only in bilateral cases but also within the same kidney containing an intra-tumoral nodule with divergent histology. While the presence of multicentric tumors in the same kidney is a recognized feature in 5%–10% of WT, in Case 13 the nodule with divergent histology was contiguous with the main tumor mass. Multicentric WT may thus be under-recognized and therefore not treated appropriately. Furthermore, under current diagnostic criteria, the relative proportions of blastemal, epithelial and stromal elements are used to stratify WT into low/intermediate/high-risk categories, with an underlying assumption that such structures are all derived from the same tumor. However, this practice needs to be refined to take into account multiple tumors, of independent origins, within the same overall mass as well as multicentric and bilateral WT.

Our findings on rarer biomarkers are more difficult to interpret in the absence of a larger multi-sampled tumor cohort. Nevertheless, our findings on 16q − highlight the importance of interpreting ITGH in the context of tumor evolution: in our series, 16q − is apparently heterogeneous only in Case 13, but it is erroneous to interpret this as evidence of 16q − ITGH, since it is present in the one sample from the smaller nodule that we showed arose independently of the remaining tumor mass. In the case of another biomarker, *MYCN* gain, we were able to relate subclonal acquisition of this change to a significantly better response to chemotherapy (as assessed on diffusion-weighted MR imaging before and after preoperative chemotherapy). *MYCN* gain may be expected to be associated with more rapid cell proliferation and therefore greater sensitivity to cytotoxic chemotherapy, and this may explain our finding. More generally, we have shown that it is feasible to integrate phylogenetic tumor analysis with monitoring of treatment response by imaging, provided that the imaging analysis is used as a guide to tumor sampling, in addition to current standard histological sampling.

Taken together, our findings in WT show unpredictable and clinically significant genetic heterogeneity that requires tumor multisampling for its detection, and assessment of tumor evolutionary trajectories for its interpretation. The custom analysis pipeline that we developed for this project may be easily applied to similar data from other multi-sampled tumors, and we are also extending it to integrate single nucleotide variants and small indels, which are typically detected in sequencing studies. Our findings have major implications for planning biomarker sampling strategies in future clinical trials for WT, and possibly other pediatric solid tumors.

## Author Contributions

GDC, JRA, BM, NJS, KPJ, RDW and WM designed the study. JRA obtained and organized clinical, radiological imaging and macrophotographic data. TC extracted DNA and carried out molecular analyses. ØEO performed radiological imaging analyses. SDP, NJS and WM reviewed histology and WM took photomicrographs. GDC, JRA, BM, CCB, MM, MEW, RDW and WM analyzed data. GDC, BM, NML and WM developed the bioinformatics analysis pipeline. NJS, KPJ, NML, RDW and WM supervised the project. GDC prepared the figures. GDC, JRA, BM, NJS, KPJ, NML, RDW and WM wrote the paper.

## Declaration of Interests

None.

## Figures and Tables

**Table 1:** Summary of patients and samples used in this study.

Case number	Gender	Age at nephrectomy (months)	Local pathological stage	Histological subtype	Number of analyzed tumor samples	Germline DNA samples analyzed (NK: non-tumorous kidney; PBL: peripheral blood lymphocytes)	ADC analysis	Additional clinical features
1	F	10	1	Stromal	2	NK, PBL	No	Contralateral nephrogenic rest
2	M	18	2	Mixed	3	None	No	Contralateral nephrogenic rest. Germline truncating *WT1* mutation in exon 7
3	M	32	3	Mixed	3	PBL	No	Large tumor complicated by hypertensive cardiomyopathy. Surgery delayed due to anesthetic risk and chemotherapy extended until surgery at 10 weeks due to tumor enlargement.
4	F	58	2	Blastemal	3	NK	No	
5	M	48	2	Regressive	2	NK	No	
6	M	19	3	Mixed	3	NK	No	Hemihypertrophy
7	F	10	2	Mixed	3	NK	No	Congenital pulmonary atresia
8	M	34	2	Mixed	3	NK	Yes	Tetralogy of fallot
9	F	32	1	Mixed	3	NK	No	Developmental delay. Constitutional gain of 22q11. 10 months before this partial nephrectomy: separate nephrogenic rest in same (left) kidney, confirmed on biopsy, and contralateral (right) regressive WT (after 9 weeks preoperative chemotherapy).
10	M	36	Right: 1 Left: 2	Right: mixed Left: mixed	Left: 2 Right: 1	NK (left)	No	Parents are first cousins
11	F	20	1	Regressive	3	NK	No	
12	F	6	1	Mixed	4	NK	No	
13	F	27	3	Mixed	5	NK	Yes	Contralateral nephrogenic rest
14	M	12	1	Mixed	2	NK	No	Incidental diagnosis of WT during investigation of bilateral cryptorchidism. Germline *WT1* substitution in exon 9 (His405Arg)
15	M	14	2	Mixed	6	NK	No	
16	M	28	Right: 3 Left: 3	Right: regressive Left: regressive	Right: 2 Left: 1	NK (right)	No	Bilateral nephron-sparing surgery with tumor at margins bilaterally
17	F	5	Right: 2 Left: 1	Right: mixed Left: mixed	Right: 3 Left: 2	NK (right)	No	
18	F	50	3	Diffuse anaplastic	3	NK	No	Tumor rupture. Pulmonary metastases.
19	F	42	1	Mixed	6	NK	No	
20	M	29	Right: 1 Left: 1	Right: mixed Left: stromal	Right: 3 Left: 2	NK (right)	No	Germline truncating *WT1* mutation in exon 9 (associated with Denys-Drash syndrome)

## Appendix A Supplementary data

Supplementary Fig. 1 Multi-sampled Wilms tumors show a variety of intra-tumor phylogenetic relationships. Here we display the phylogenetic trees of all 17 cases for which a phylogenetic tree was generated using our analysis. Nodes represent samples where green nodes are normal tissue and red nodes are tumor. Each node is annotated with its region number and in the case of bilateral tumors this is preceded by an R or and L denoting the right and left kidney respectively. Black edges are weighted by number of events vertically and are used to separate samples horizontally. The scale for the number of events in denoted to the left of each phylogenetic tree in each case. Supplementary Fig. 2 Chromosome 11p copy number neutral loss of heterozygosity (CNNLOH) is an early driver event in Wilms tumorigenesis. Each plot represents the B-allele frequency data for cases in which one sample contains a chromosome 11p copy number neutral CNNLOH event. For each plot chromosome 11 position (MB) is displayed on the x-axis and the B-allele frequency is displayed on the y-axis. The first row of plots shows the normal tissue sample for each case (a normal sample was not available for Case 2) and the cases are ordered in columns. The remaining B-allele frequency plots from the second row onwards are tumor samples for each case. Points in blue represent probes called as being in a state of loss of heterozygosity by our algorithm. The orange box in the first tumor sample of Case 14 represents a region of CNNLOH in chromosome 11p not detected by our algorithm. The green box in the fourth and fifth tumor sample in Case 20 represents the centromeric boundary difference of the copy number neutral loss of heterozygosity event in the left kidney samples compared to the right kidney samples (tumor samples 1–3). Supplementary Fig. 3 Chromosome 1q gain (1q +) is heterogeneous in Wilms tumors. Each plot represents the Log R ratio data of all samples from cases with at least one case that has 1q +. Chromosome 1 position (MB) is displayed on the x-axis and the Log R ratio value on the y-axis. The first row contains all normal tissue samples for each case which are ordered by column, with unilateral Wilms tumors grouped on the left and bilateral Wilms tumors displayed on the right. Log R ratio plots displayed from the second row onwards are all tumor samples. Bilateral Wilms tumor samples are further grouped by the kidney of origin (left and right kidney respectively). Log R ratio values called as gained by CGHcall are displayed in green with the background of the plot coloured green if the sample shows 1q +. Supplementary Fig. 4 Representative illustration of MS-MLPA analysis in tumors with 11p15 CNNLOH. (A) Upper part: Tumor sample from Case 20 showing copy number calculation within normal limits for 11p15 probes as well as reference probes. Lower part: calculated methylation levels for each probe, with expected normal range between blue and red lines. (B) Equivalent analysis carried out on a normal kidney sample. Image 1

Supplementary material 1 Image 2

Supplementary material 2 Image 3

Supplementary material 3 Image 4
