# Supplementary material for: Intra-Tumor Genetic Heterogeneity in Wilms Tumor: Clonal Evolution and Clinical Implications
Source: eBioMedicine. 2016 May 27;9:120–9. doi: 10.1016/j.ebiom.2016.05.029 (PMC4972528; doi:10.1016/j.ebiom.2016.05.029)

Supplementary Figure 1

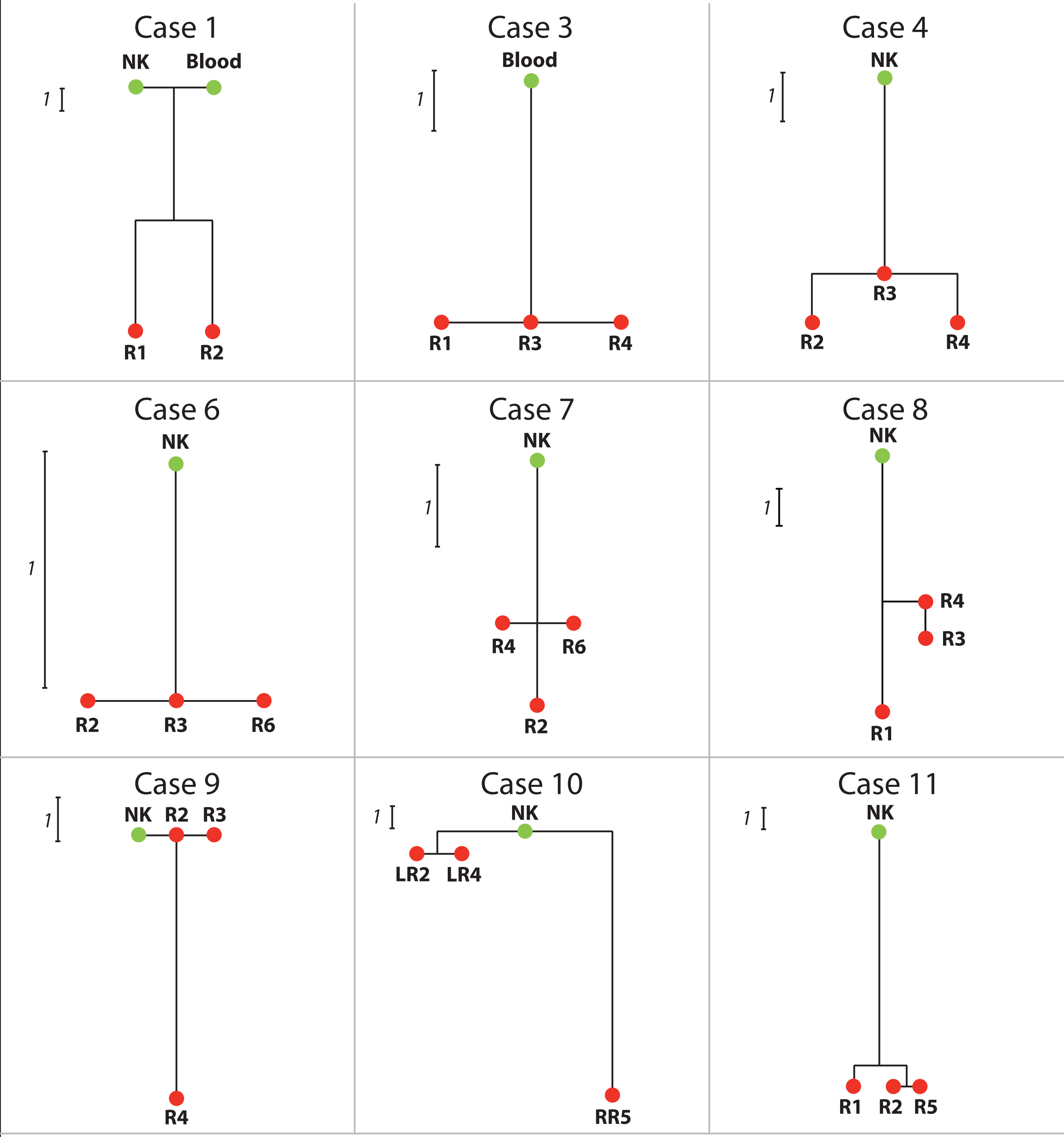

Supplementary Figure 1 (continued)

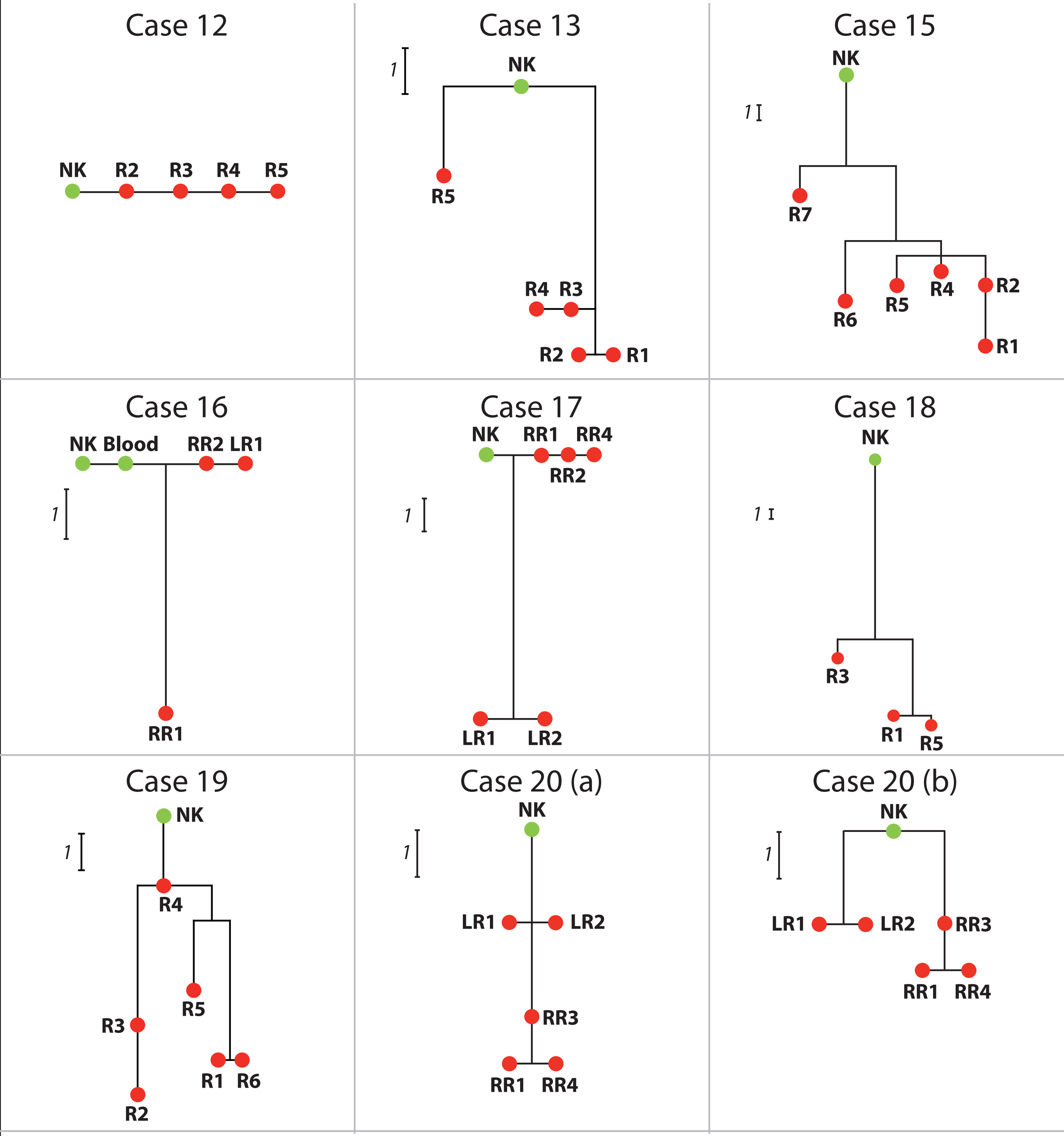

Supplementary Figure 2

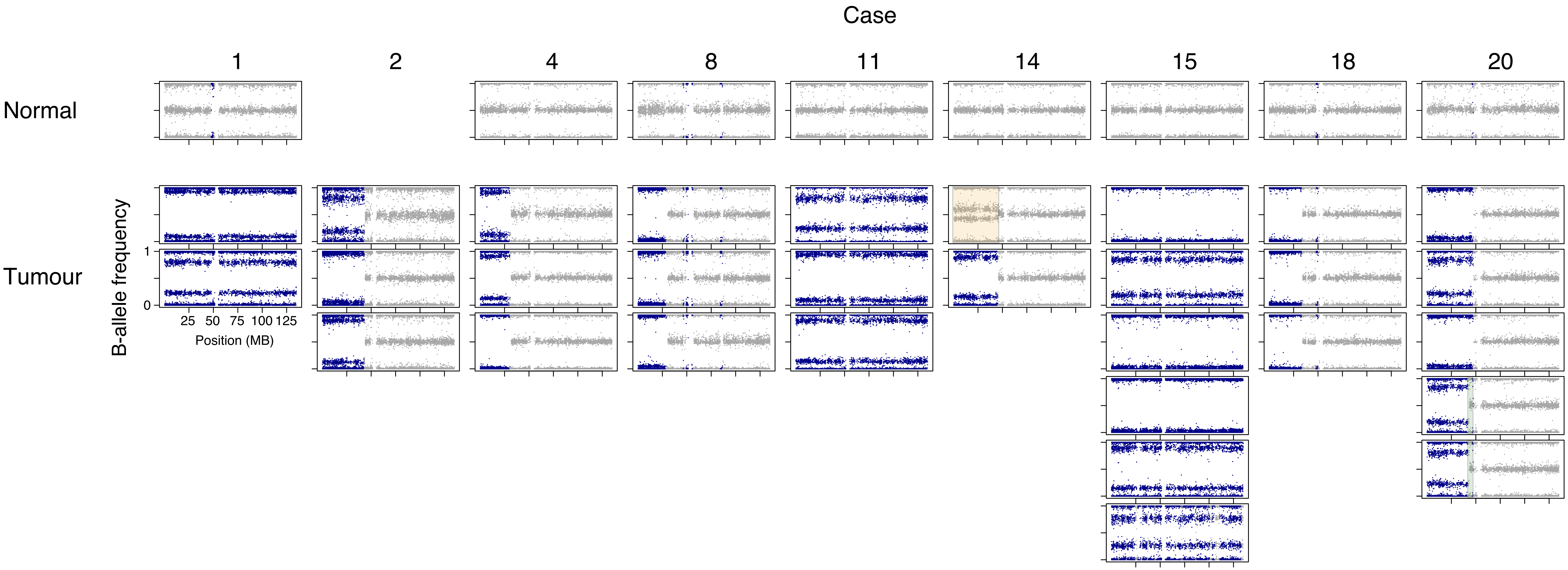

Supplementary Figure 3

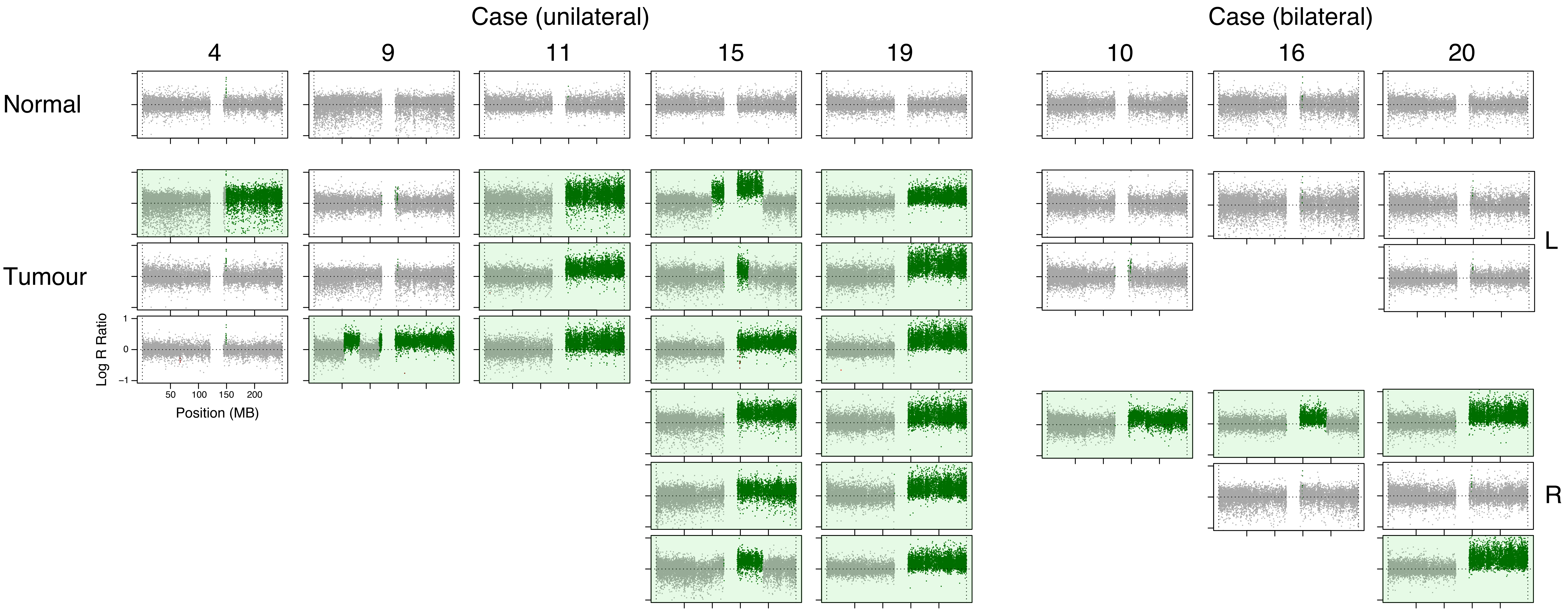

Supplementary Figure 4

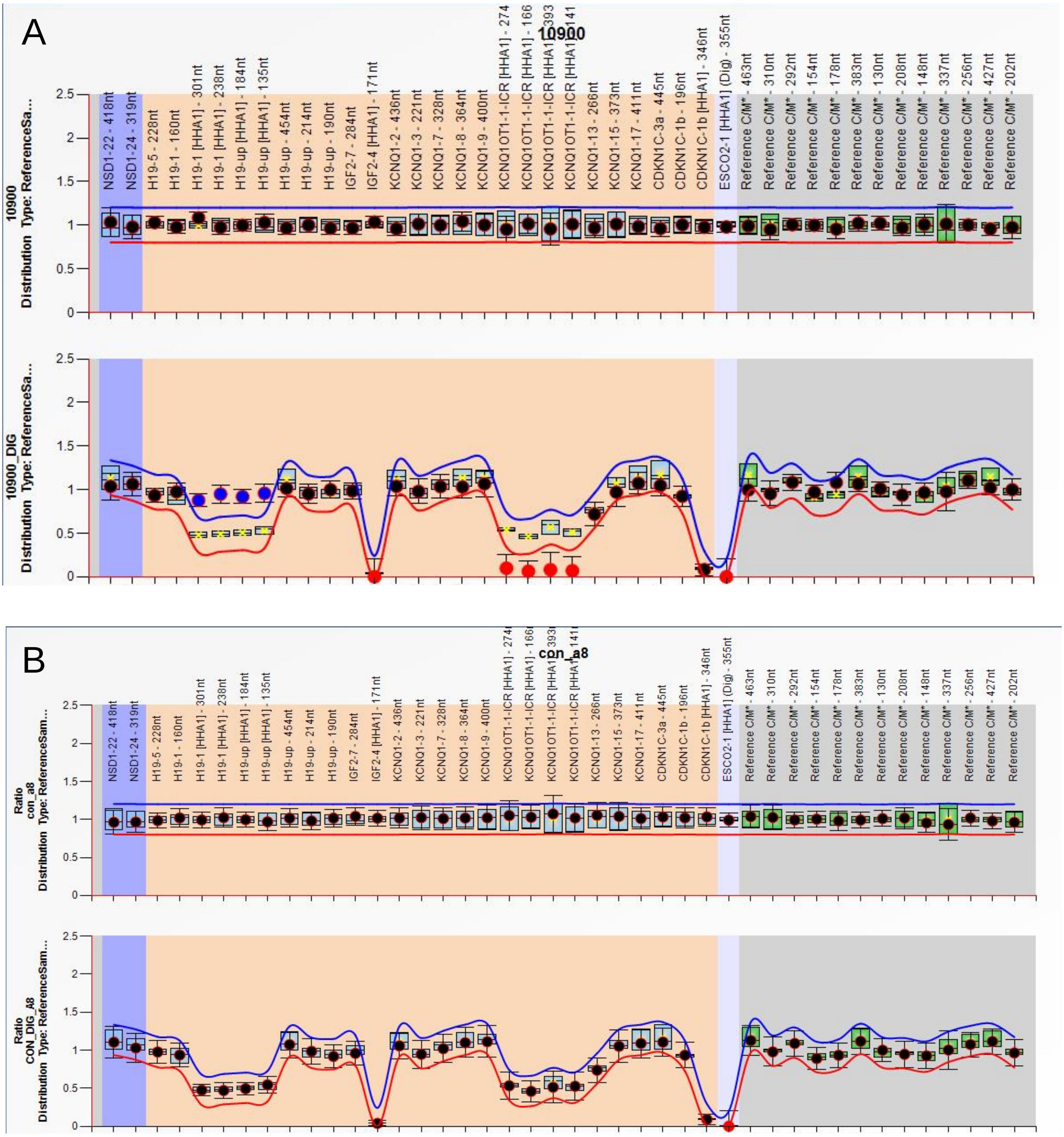

Supplement: Supplementary file 1 — Supplementary Fig. 1 Multi-sampled Wilms tumors show a variety of intra-tumor phylogenetic relationships. Here we display the phylogenetic trees of all 17 cases for which a phylogenetic tree was generated using our analysis. Nodes represent samples where green nodes are normal tissue and red nodes are tumor. Each node is annotated with its region number and in the case of bilateral tumors this is preceded by an R or and L denoting the right and left kidney respectively. Black edges are weighted by number of events vertically and are used to separate samples horizontally. The scale for the number of events in denoted to the left of each phylogenetic tree in each case. Supplementary Fig. 2 Chromosome 11p copy number neutral loss of heterozygosity (CNNLOH) is an early driver event in Wilms tumorigenesis. Each plot represents the B-allele frequency data for cases in which one sample contains a chromosome 11p copy number neutral CNNLOH event. For each plot chromosome 11 position (MB) is displayed on the x-axis and the B-allele frequency is displayed on the y-axis. The first row of plots shows the normal tissue sample for each case (a normal sample was not available for Case 2) and the cases are ordered in columns. The remaining B-allele frequency plots from the second row onwards are tumor samples for each case. Points in blue represent probes called as being in a state of loss of heterozygosity by our algorithm. The orange box in the first tumor sample of Case 14 represents a region of CNNLOH in chromosome 11p not detected by our algorithm. The green box in the fourth and fifth tumor sample in Case 20 represents the centromeric boundary difference of the copy number neutral loss of heterozygosity event in the left kidney samples compared to the right kidney samples (tumor samples 1–3). Supplementary Fig. 3 Chromosome 1q gain (1q +) is heterogeneous in Wilms tumors. Each plot represents the Log R ratio data of all samples from cases with at least one case that has 1q +. [file mmc1.pdf]
