# Supplementary material for: Intra-Tumor Genetic Heterogeneity in Wilms Tumor: Clonal Evolution and Clinical Implications
Source: eBioMedicine. 2016 May 27;9:120–9. doi: 10.1016/j.ebiom.2016.05.029 (PMC4972528; doi:10.1016/j.ebiom.2016.05.029)
Supplement: Supplementary file 2 — Supplementary material 1 [file mmc2.zip › CGH_pipeline_documentation.html]

CGH pipeline: Generation of genomic regions of intratumour copy number changes for phylogenetic analysis


# CGH pipeline: Generation of genomic regions of intratumour copy number changes for phylogenetic analysis

The purpose of this analysis is to use multiple tumour region SNP arrays and a normal tissue SNP array to infer phylogenetic relationships of these multiple tumour regions relative to the normal sample and each other, and to consequentially assess intratumour heterogeneity and clonal evolution.

Therefore the only raw data needed in this analysis is the B-allele frequency and the Log R ratio data from each array. The pipeline also requires multiple freely available libraries and a pipeline specific source file. This example will run through one case.

We begin by loading all our LRR and BAF data and selecting our case of interest. Which will be case=16.

```
case=16

#Raw data
load("~/Wilms_tumour_project/results/2014-06-09/BAFreqs_all.Rdata")
load("~/Wilms_tumour_project/results/2014-06-09/LogR_Ratios_all_v2.Rdata")

#Case specific data
case.LRR <- LogR_Ratios_all[[case]]
case.BAF <- BAFreqs_all[[case]]
```

The LRR and BAF data is stored as a dataframe that represents a patient. In this dataframe the first three columns are:

1. “Name” - The SNP probe name, e.g. rs123456.
2. “Chr” - Chromosome number.
3. “Position” - The position on the chromosome in base pairs of the SNP probe.

Columns 4 to *n* are either the LRR or BAF values for each SNP probe for each array of a single patient (where *n* is the number of arrays).

```
head(case.LRR, n=3)
```

```
##        Name Chr Position X10998.Log.R.Ratio X11000.Log.R.Ratio
## 1 rs3094315   1   752566       -0.182194400        -0.06591658
## 2 rs2073813   1   753541       -0.217497600        -0.03981428
## 3 rs2905040   1   770216        0.005371154        -0.12572690
##   X11002.Log.R.Ratio X10999.Log.R.Ratio X11001.Log.R.Ratio
## 1        -0.05061141        -0.05349346        -0.03555601
## 2         0.04952753        -0.12626220        -0.02510486
## 3        -0.04830415        -0.11189390        -0.16129360
##   X11003.Log.R.Ratio X11004.Log.R.Ratio
## 1        -0.07229545       -0.062912330
## 2        -0.12662270        0.007890958
## 3        -0.12833280       -0.219372500
```

```
head(case.BAF, n=3)
```

```
##        Name Chr Position X10998.B.Allele.Freq X11000.B.Allele.Freq
## 1 rs3094315   1   752566         0.0007238651              0.00000
## 2 rs2073813   1   753541         0.9855661000              0.98374
## 3 rs2905040   1   770216         1.0000000000              1.00000
##   X11002.B.Allele.Freq X10999.B.Allele.Freq X11001.B.Allele.Freq
## 1            0.0000000            0.0000000            0.0000000
## 2            0.9528209            0.9937303            0.9791941
## 3            0.9935530            1.0000000            0.9951853
##   X11003.B.Allele.Freq X11004.B.Allele.Freq
## 1            0.0000000            0.0000000
## 2            0.9990678            0.9958999
## 3            0.9976220            0.9999461
```

The following plots represent the LRR data for chromosome 1 in our case. Notice the variable genomic waviness, particularly on the q-arm (which is gained in samples 1-6).

The following plots represent the BAF data for chromosome 1 in our case. B-allele frequency data is not affected by genomic waviness as the phenomena does not show any allelic bias.

In order to use functions from freely available software for our analysis, we must load these libraries.

```
library("CGHcall")
library("CGHregions")
library(seqinr)
library(plyr)
```

We must also define some parameters for our analysis:

The first three parameters are related to the settings used for segmentation in the CGHcall package.

1. *undo.SD* - A split generating two segments is undone according to CGHcall’s *undo.splits* setting if their mean LRR is less than this number of standard deviations apart, when involving at least one ‘small’ segment.
2. *clen* - Segments shorter than *clen* probes are considered ‘small’ and segments greater in size are considered ‘long’ by CGHcall.
3. *relSDlong* - *undo.SD* / *relSDlong* is the number of standard deviations used for undoing splits for two ‘long’ segments.
4. *crit* - New region boundaries are introduced by CGHregions if more than this number of arrays has a different copy number state as the algorithm assesses the chromosome. Default = 0, as this means all copy number changes are used to generate regions.
5. *iqr.threshold* - Regions are removed if their density (bp SNP-1) are ± *iqr.threshold* \* IQR of all measured inter-quartile ranges in the dataset. However, in this example we will not use this as we will only assess one case, instead we will use the limits on density calculated from all cases in this investigation (3162 bp snp-1 - 25119 bp snp-1).
6. *snp.threshold* - This is the minimum number of SNPs needed in a region for it to be used in phylogenetic analysis.

```
undo_SD       <- 5
clen          <- 13
relSDlong     <- undo_SD*(5/3)
crit          <- 0
iqr_threshold <- 2
snp_threshold <- 100
```

The first step in the analysis is to process the BAF by converting it to mirrored-BAF and subsequently removing the non-informative homozygous SNPs and scaling the remaining SNPs to produce a tumour specific profile. For this we use estimations of aberrant cell fraction generated by ASCAT (http://heim.ifi.uio.no/bioinf/Projects/ASCAT/). These estimations are a minimum of 0.8 and a maximum of 0.95. The 0.95 maximum is used here, but not later, as higher values will not generate an upper threshold that will remove non-informative homozygous SNPs.

The BAF is mirrored using the following equation:

> (| B - 0.5 |) + 0.5 = M

Where:

1. B is the BAF.
2. M is the mBAF.

In this investigation we do not analyse chromosome Y as it often produces many aberrant copy number calls and only occurs in male samples. Chromosome X is renamed chromosome 23 to allow for it to be manipulated as a number.

```
#Aberrant cell fractions used to process the BAF
ASCAT.mBAF <- c(0.91, 0.93, 0.8, 0.8, 0.82, 0.95, 0.95)

#Convert BAF to mBAF
mBAF.matrix <- abs(case.BAF[,4:ncol(case.BAF)] - 0.5) + 0.5
case.mBAF <- cbind(case.BAF[,1:3], mBAF.matrix)

head(case.mBAF, n=2)
```

```
##        Name Chr Position X10998.B.Allele.Freq X11000.B.Allele.Freq
## 1 rs3094315   1   752566            0.9992761              1.00000
## 2 rs2073813   1   753541            0.9855661              0.98374
##   X11002.B.Allele.Freq X10999.B.Allele.Freq X11001.B.Allele.Freq
## 1            1.0000000            1.0000000            1.0000000
## 2            0.9528209            0.9937303            0.9791941
##   X11003.B.Allele.Freq X11004.B.Allele.Freq
## 1            1.0000000            1.0000000
## 2            0.9990678            0.9958999
```

```
#Make X numeric
case.mBAF$Chr <- revalue(case.mBAF$Chr, c("X"="23"))
case.mBAF$Chr <- as.numeric(as.character(case.mBAF$Chr))

#Remove the NAs that come from the Y chromosome
case.mBAF <- case.mBAF[which(!is.na(case.mBAF$Chr)),]

#Order the dataframe by chromosome and then position
case.mBAF <- case.mBAF[with(case.mBAF, order(Chr, Position)), ]

#Find and remove duplicated SNP probes
pos <- paste(case.mBAF$Chr,case.mBAF$Position,sep='_')
ss <- which(duplicated(pos))
s <- which(pos%in%pos[ss])
case.mBAF <- case.mBAF[-s,]
```

For each sample array (*s*), the ASCAT generated aberrant cell fraction is used to calculate an *upper.threshold* above which SNPs are considered to be homozygous, and therefore non-informative. Using the equation:

> 1 / (1 + (1 - A)) = T

Where:

1. A is the aberrant cell fraction.
2. T is the upper threshold.

Non-informative homozygous SNPs are replaced with NAs. The remaining SNPs are normalised linearly to the upper threshold if the mBAF value is greater than 0.56. This is because heterozygous diploid SNPs (mBAF <= 0.56) are not different between the tumour and the contaminating normal cells, however copy number changes that result in allelic imbalance are ‘diluted’ by the normal cell contamination. Therefore scaling attempts to reconstruct the allelic imbalance present only in the cancer cells.

```
for(s in 1:(ncol(case.mBAF)-3)) {
      #Use the sample specific aberrant cell fraction
      ACF <- ASCAT.mBAF[s]
      
      #Calculate the upper threshold for removing homozygous SNPs
      upper.threshold <- 1 / (1+(1-ACF))
      
      #Make homozygous SNPs = NA
      case.mBAF[which(case.mBAF[,s+3] > upper.threshold),s+3] <- NA
      
      #Scale the remaining SNPs if greater than 0.56
      case.mBAF[which(case.mBAF[,s+3] > 0.56),s+3] <- (0.5*((case.mBAF[which(case.mBAF[,s+3] > 0.56),s+3]-0.5)/(upper.threshold-0.5)))+0.5
    }
```

The following plots represent the mBAF data for chromosome 1 in our case. Scaling begins after 0.56. This is a tumour specific profile.

For the rest of the analysis we will need the source file specific to this pipeline.

```
source("~/Wilms_tumour_project/bin/Original_George_Scripts/CGH_pipeline_source_file_v2.R")
```

We also need to load the genomic waviness profile generated previously. This means loading the profiles for the autosomes, male and female X chromosomes separately and combining them on for each sex.

```
#Load genomic waviness
load("~/Wilms_tumour_project/results/2014-09-25/movav_avnorm_genome.Rdata")
load("~/Wilms_tumour_project/results/2015-01-29/female.chr.X.movav.Rdata")
load("~/Wilms_tumour_project/results/2015-01-29/male.chr.X.movav.med.zeo.Rdata")

#Make gender specific genomic waviness
female.movav_avnorm_genome <- c(movav_avnorm_genome, female.chr.X.movav)
male.movav_avnorm_genome   <- c(movav_avnorm_genome, male.chr.X.movav.med.zeo)
```

Firstly, we’ll make an output directory for this particular case and save the original LRR data in it.

```
#Save raw LRR data
system(paste0("mkdir ",names(LogR_Ratios_all)[case]))
save(case.LRR, file=paste0(names(LogR_Ratios_all)[case],"/",names(LogR_Ratios_all)[case],"_LogR_ratios_raw.Rdata"))
```

Next we remove the Y chromosome, re-define X as 23 and order the rows by chromosome and then position. It is therefore in the same order as the genomic waviness object.

```
#Make X numeric
case.LRR$Chr <- revalue(case.LRR$Chr, c("X"="23"))
case.LRR$Chr <- as.numeric(as.character(case.LRR$Chr))

#Remove the NAs that come from the Y chromosome
case.LRR <- case.LRR[which(!is.na(case.LRR$Chr)),]

#Order the dataframe by chromosome and then position
case.LRR <- case.LRR[with(case.LRR, order(Chr, Position)), ]
```

The genomic waviness profile (shown in blue) maps the bias present across chromosome 1.

As the normalisation is performed differently for each gender we must input the *sex.info*. This case is female.

```
#This case is female
sex.info <- NULL
sex.info[case] <- "F"

if(sex.info[case]=="F") {
    for (array in 4:ncol(case.LRR)) {
      
      #Remove genomic waviness for female
      case.LRR[,array] <- movav_normalise(case.LRR[,array], female.movav_avnorm_genome)
    }
    
    #Save the normalised LRR data
    save(case.LRR, file=paste0(names(LogR_Ratios_all)[case],"/",names(LogR_Ratios_all)[case],"_LogR_ratios_normalised.Rdata"))
    }
```

```
## final weight is: 0.9 
## final weight is: 1 
## final weight is: 0.9 
## final weight is: 1.4 
## final weight is: 1.3 
## final weight is: 1.1 
## final weight is: 0.4
```

```
if(sex.info[case]=="M") {
    for (array in 4:ncol(case.LRR)) {
        
      #Remove genomic waviness for male
      case.LRR[,array] <- movav_normalise(case.LRR[,array], male.movav_avnorm_genome)
    }
      
    #Save the normalised LRR data
    save(case.LRR, file=paste0(names(LogR_Ratios_all)[case],"/",names(LogR_Ratios_all)[case],"_LogR_ratios_normalised.Rdata"))
    }
```

Genomic waviness has now been removed from the Log R ratio data. Notice how all arrays are now relatively straightened out.

Next we prepare the Log R ratio dataframe for input into the CGHcall software.

```
#Find and remove duplicated SNP probes
pos <- paste(case.LRR$Chr,case.LRR$Position,sep='_')
ss <- which(duplicated(pos))
s <- which(pos%in%pos[ss])
case.LRR <- case.LRR[-s,]

#Preprare CGHcall object
case.LRR$end <- case.LRR$Position
case.LRR <- case.LRR[c(1:3,length(case.LRR),4:(length(case.LRR)-1))]
colnames(case.LRR)[1]='BAC.clone'
colnames(case.LRR)[2]='CHROMOSOME'
colnames(case.LRR)[3]='START_POS'
colnames(case.LRR)[4]='END_POS'
case.LRR <- na.omit(case.LRR)
```

Next we perform the CGHcall protocol using aberrant cell fractions from ASCAT for cellularity. These aberrant cell fractions do not have an upper cap of 0.95 needed to estimate the upper threshold and may be greater than 0.95, and indeed, 1.

```
#CGHcall specific aberrant cell fractions
ASCAT.CGHcall <- c(0.91, 0.93, 0.8, 0.8, 0.82, 1, 1)

#CGHcall processing
case.LRR <- make_cghRaw(case.LRR)
cghdata_case <- preprocess(case.LRR, maxmiss = 0, nchrom = 23)
norm.case.LRR <- normalize(cghdata_case, method="median", smoothOutliers=TRUE)
seg.case.LRR <- segmentData(norm.case.LRR, method = "DNAcopy", undo.splits = "sdundo", undo.SD = undo_SD, clen = clen, relSDlong = relSDlong)
postseg.case.LRR <- postsegnormalize(seg.case.LRR)
result <- CGHcall_edit(postseg.case.LRR, nclass = 5, cellularity = ASCAT.CGHcall)
case.LRR.Called <- ExpandCGHcall(result, postseg.case.LRR)

#Save CGHcall output
save(case.LRR.Called, file=paste0(names(LogR_Ratios_all)[case],"/",names(LogR_Ratios_all)[case],"_CGHcall_output.Rdata"))
```

The following plots show the calls for chromosome 1 overlayed ontop of the Log R ratio data.

The following plots show the calls for all probes in Sample 1 overlayed ontop of the Log R ratio data.

The next step involves calculating the genomic regions of copy number changes using CGHregions.

```
#Perform CGHregions
case_regions_c <- CGHregions_GC_edit(case.LRR.Called, critfound=crit)

#Save CGHregions output
save(case_regions_c, file=paste0(names(LogR_Ratios_all)[case],"/",names(LogR_Ratios_all)[case],"_CGHregions_output.Rdata"))
```

The following plot represents the regions of copy number changes across all samples. Where chromosomes are represented on the y-axis and a new region boundary is displayed as a shift up/down along the chromosome position. Colours represent the mean copy number call in these regions across the samples, where green is an average copy number gain and red is an average copy number loss, black represents an average copy number call of 0. Brighter colours indicate higher number of gains/losses on average.

Next, we will filter calls by ensuring the mBAF values correspond to the calls.

```
#Convert CGHregions object to a dataframe
regions_dataframe <- regions.as.table(case_regions_c)

#Calculate mean mBAF value per genomic region
case_bafs <- mean_mBAF_per_region(regions_dataframe, case.mBAF, chr_num=23)

#Revert call to zero if mBAF does not achieve the threshold for gains, loss and amplifications
case_list <- mBAF_based_gain_QC(regions_dataframe, case_bafs)
case_list <- mBAF_based_loss_QC(case_list[[1]], case_list[[2]])
case_list <- mBAF_based_amp_QC(case_list[[1]], case_list[[2]])
regions_dataframe <- case_list[[1]]
```

Next we calculate some simple descriptive statistics regarding our genomic regions.

```
#Create a dataframe including all SNPs and calls
SNPcalls_dataframe <- cbind(chromosomes(case.LRR.Called),
                            bpstart(case.LRR.Called),
                            calls(case.LRR.Called))

#Count the number of SNPs present in each genomic region
SNPs_no <- NULL
for (n in 1:nrow(regions_dataframe)) {
   SNPS <- SNPcalls_dataframe[which(SNPcalls_dataframe[,1]==regions_dataframe[n,1] 
                                       & SNPcalls_dataframe[,2] >= regions_dataframe[n,2]
                                       & SNPcalls_dataframe[,2] <= regions_dataframe[n,3]) , ]
   row <- nrow(SNPS)
   SNPs_no <- rbind(SNPs_no, row)
}
rownames(SNPs_no) <- NULL

#Add number of SNPs to regions dataframe
regions_dataframe_SNPs <- data.frame(regions_dataframe[,1:3], SNPs_no, regions_dataframe[,4:ncol(regions_dataframe)])
colnames(regions_dataframe_SNPs)[1:4] <- c("Chr", "Start", "End", "SNPs_no")

#Calculate widths of regions
width <- regions_dataframe_SNPs[,"End"]-regions_dataframe_SNPs[,"Start"]

#Calculate densities of regions
bp_per_SNP <- width/regions_dataframe_SNPs[,"SNPs_no"]

#Add densities to dataframe
regions_dataframe_SNPs <- data.frame(regions_dataframe_SNPs[,1:4], bp_per_SNP, regions_dataframe_SNPs[,5:ncol(regions_dataframe_SNPs)])
```

As we are only running through a single case, we will use the density thresholds calculated from the dataset as a whole instead of recalculating using \_iqr.threshold\_xIQR on a single case as this will result in slightly different boundaries. Therefore we will use *upper* = 4.4 and *lower* = 3.5, these are used as an exponent with the base of 10.

```
upper <- 4.4
lower <- 3.5
```

Remove regions that are too small or have extreme density.

```
#Filter out regions that are not large enough
regions_dataframe_SNPs_sxrm_snpflt <- regions_dataframe_SNPs[regions_dataframe_SNPs[,"SNPs_no"]>snp_threshold,]

#Filter out regions with extreme density
regions_dataframe_SNPs_sxrm_snpflt_dnsflt <- regions_dataframe_SNPs_sxrm_snpflt[which(regions_dataframe_SNPs_sxrm_snpflt[,"bp_per_SNP"]<(10^upper) & regions_dataframe_SNPs_sxrm_snpflt[,"bp_per_SNP"]>(10^lower)),]

#Save these final regions
save(regions_dataframe_SNPs_sxrm_snpflt_dnsflt, file=paste0(names(LogR_Ratios_all)[case],"/",names(LogR_Ratios_all)[case],"_final_filtered_regions.Rdata"))
```

The following plot represents the final regions that have been filtered for size, density and calls.

Finally we calculated the major and minor allele copy numbers using the mBAF and the total copy number for each genomic region, using the following equations:

> round(T \* M) = A

> T - A = B

Where:

1. T = Total copy number of region
2. M = Mean mBAF of region
3. A = Major allele copy number
4. B = Minor allele copy number

The major and minor allele copy numbers are outputted as FASTA files that are compatible with MEDICC.

```
#Calculate mean mBAF per region
case_mean_mBAF_per_regions <- mean_mBAF_per_region(regions_dataframe_SNPs_sxrm_snpflt_dnsflt, case.mBAF, chr_num=23)
colnames(case_mean_mBAF_per_regions) <- c("Chr", "Start", "End", sampleNames(case.LRR.Called))

rownames(regions_dataframe_SNPs_sxrm_snpflt_dnsflt) <- NULL

#Add 2 to make total copy number
copynumber <- regions_dataframe_SNPs_sxrm_snpflt_dnsflt[,6:ncol(regions_dataframe_SNPs_sxrm_snpflt_dnsflt)]+2
mBAF_per_region_sub <- case_mean_mBAF_per_regions[,4:ncol(case_mean_mBAF_per_regions)]

#Calculate major and minor copy number using mBAF
Major_A <- round(copynumber*mBAF_per_region_sub)
Minor_A <- copynumber-Major_A

#Vector of chromosomes
C <- regions_dataframe_SNPs_sxrm_snpflt_dnsflt[,"Chr"]

#Format the major allele copy numbers as a list
chrom_major <- cbind(C,Major_A)    
PerChrom <- list()
length(PerChrom) <- max(C)
for(l in 1:max(C)){
  PerChrom[[l]] <- subset(chrom_major,C==l)
}
MajorAppChrom <- PerChrom

#Format the minor allele copy numbers as a list
chrom_minor <- cbind(C,Minor_A)
PerChrom <- list()
length(PerChrom) <- max(C)
for(l in 1:max(C)){
  PerChrom[[l]] <- subset(chrom_minor,C==l)
}
MinorAppChrom <- PerChrom

#Record number of samples in this case
num_samples <- ncol(regions_dataframe_SNPs_sxrm_snpflt_dnsflt)-5

#Make output directory for FASTA output
system(paste0("mkdir ",names(BAFreqs_all[case]),"_FASTA"))

#Write minor allele FASTA file for each chromosome
for (u in 1:max(C)){
  for (s in 1:num_samples) {
    write.fasta(MinorAppChrom[[u]][,s+1], names=paste0(colnames(case.mBAF[s+3])), nbchar=50, file.out=paste0(names(BAFreqs_all[case]),"_FASTA/Minor_chr",u,".fasta"), open="a")
  }
}

#Write major allele FASTA file for each chromosome
for (u in 1:max(C)){
  for (s in 1:num_samples) {
    write.fasta(MajorAppChrom[[u]][,s+1], names=paste0(colnames(case.mBAF[s+3])), nbchar=50, file.out=paste0(names(BAFreqs_all[case]),"_FASTA/Major_chr",u,".fasta"), open="a")
  }
}
```
