# Supplementary material for: Intra-Tumor Genetic Heterogeneity in Wilms Tumor: Clonal Evolution and Clinical Implications
Source: eBioMedicine. 2016 May 27;9:120–9. doi: 10.1016/j.ebiom.2016.05.029 (PMC4972528; doi:10.1016/j.ebiom.2016.05.029)
Supplement: Supplementary file 3 — Supplementary material 2 [file mmc3.zip › CNN_LOH_detection_algorithm.html]

Detection of copy number neutral loss-of-heterozygosity regions


# Detection of copy number neutral loss-of-heterozygosity regions

Begin by sourcing function from the CGH pipeline source file and load up the mirrored B-allele frequency (mBAF) data that has been processed by:

1. Removal of non-informative homozygous SNPs (and replaced with ‘NA’).
2. Scaling of remaining SNPs.

The mBAF data is a list in which each element in the list is a different patient. The element of the patient then contains a dataframe in which the first three columns are:

1. “Name” - The SNP probe name, e.g. rs123456.
2. “Chr” - Chromosome number, where X = 23.
3. “Position” - The position on the chromosome in base pairs of the SNP probe.

Columns 4 to n are the mBAF values for each SNP probe for each array of a single patient.

The algorithm will work with non-processed mBAFs, but aberrant cell fraction must be considered when interpretting results. Furthermore, the removal of non-informative homozygous SNPs is not necessary as they are not used in the analysis. It must however be mBAF and not BAF.

We will use case=9 as an example in this documentation as it contains two chromosome 2 copy number neutral LOH events.

```
case = 9
```

```
#Source file with the functions we need for this investigation
source("~/Wilms_tumour_project/bin/Original_George_Scripts/CGH_pipeline_source_file_v2.R")

#Load the processed mBAF
load("~/Wilms_tumour_project/results/2015-03-10/Threshold_mBAF_v3.Rdata")

#Record number of samples in this case
n.samples      <- ncol(Threshold_mBAF_v3[[case]]) - 3
head(Threshold_mBAF_v3[[case]])
```

```
##         Name Chr Position X8890.B.Allele.Freq X8891.B.Allele.Freq
## 1  rs3094315   1   752566           0.5432649           0.5409348
## 2  rs2073813   1   753541           0.5427273           0.6168932
## 3  rs2905040   1   770216                  NA                  NA
## 4 rs12124819   1   776546                  NA                  NA
## 5  rs2980314   1   781258                  NA                  NA
## 6  rs6684487   1   791853                  NA                  NA
##   X8892.B.Allele.Freq X8893.B.Allele.Freq
## 1           0.5440590           0.5296163
## 2           0.5290127           0.6725110
## 3                  NA                  NA
## 4                  NA                  NA
## 5                  NA                  NA
## 6                  NA                  NA
```

These plots show the raw data in the form of processed mBAF data.

These are the packages required for the analysis:

```
library(GenomicRanges)
library(plyr)
library(CGHcall)
library(seqinr)
```

These are the parameters used for detecting the copy number neutral changes and subsequently merging the detected regions so they are interpretable in the context of the regions detected from the Log R ratio data.

The parameters are as follows:

1. *window* - This is the size of the sliding window in which the number of SNPs in the ‘AB state’ is counted. This window slides by 10 SNPs across the chromosome and recounts the SNPs. Default = 100 SNPs.
2. *AB.state.threshold* - The ‘AB state threshold’ is the value at which mBAF values which are less than or equal are considered to be in the ‘AB state’. The default is mBAF = 0.66. This is chosen as copy number gains result in mBAF = 0.66 but are not copy number neutral shifts from the AB state. Therefore these changes will not be detected by this algorithm as changes from the AB state. However, loss-of-heterozygosity, which results in mBAF ~ 0.95 will be detected (both copy number neutral and copy number loss). More complex allelic ratios may also be detected (e.g. strong amplifications).
3. *loh.threshold* - The number of SNPs in the ‘AB state’ is calculated as a percentage of window size. If the number is less than or equal to this value, it is called as being a loss of heterozygosity. The default is 1% of the size of the window.
4. *con.bins.merge* - As each array is analysed individually but are often likely to share LOH events, the boundaries may possibly be determined slightly differently in each array, despite, in all likelihood being the same. Therefore we choose to smooth these boundaries and make them consistent between arrays. Therefore we smooth boundaries together if they are less than or equal to *con.bins.merge* bins apart. Default = 10 bins.
5. *region.merger* - Genomic regions generated from the Log R ratio are removed if they are less than 100 SNPs in size. In order to prevent this analysis generating regions that are smaller than 100 SNPs, we use *region.merger* to merge regions less than 100 SNPs to a neighbour, to essentially ensure regions are never smaller than 100 SNPs and consistent with the Log R ratio regions. This should reflect the threshold of size filtering of the Log R ratio regions (and if the Log R ratios are not filtered for size, *region.merger* = 0).
6. *LOH.size.threshold* - This was introduced to only take large CNN LOH regions, as we were only particularly interested in large regions for generating the phylogenetic trees. This can be used as a final size filter at the end of the analysis. We use *LOH.size.threshold* = 1000.

```
window=100
AB.state.threshold = 0.66
loh.threshold = window*0.01
con.bins.merge = 10
region.merger = 100 
LOH.size.threshold = 1000
```

```
chr.vector = 1:23
```

Move into the working directory that contains the output of the main Log R ratio regions analysis and load the CGHcall output for information on the SNPs that were analysed in this case and load the genomic regions of copy number changes determined at the level of the Log R ratio data. We will use the ‘final filtered regions’ as a reference for genomic regions that may be missing the detection of CNN LOH.

```
#Load necessary output from the CGH pipeline
load(paste0(names(Threshold_mBAF_v3)[case],"/",names(Threshold_mBAF_v3)[case],"_CGHcall_output.Rdata"))
load(paste0(names(Threshold_mBAF_v3)[case],"/",names(Threshold_mBAF_v3)[case],"_final_filtered_regions.Rdata"))

case_LogR_ratioCalled
```

```
## cghCall (storageMode: lockedEnvironment)
## assayData: 293831 features, 4 samples 
##   element names: calls, copynumber, probamp, probdloss, probgain, probloss, probnorm, segmented 
## protocolData: none
## phenoData
##   sampleNames: X8890.Log.R.Ratio X8891.Log.R.Ratio
##     X8892.Log.R.Ratio X8893.Log.R.Ratio
##   varLabels: Cellularity
##   varMetadata: labelDescription
## featureData
##   featureNames: rs2980314 rs6684487 ... rs553678 (293831 total)
##   fvarLabels: Chromosome Start End
##   fvarMetadata: labelDescription
## experimentData: use 'experimentData(object)'
## Annotation:
```

```
head(regions_dataframe_SNPs_sxrm_snpflt_dnsflt)
```

```
##    Chr     Start       End SNPs_no bp_per_SNP X8890.Log.R.Ratio
## 1    1    781258 120512303   13020   9195.933                 0
## 3    1 145388137 147828939     470   5193.196                 0
## 7    1 149815079 249162263    9426  10539.697                 0
## 8    2    106409  15619986    2330   6658.188                 0
## 9    2  15626327  16445097     132   6202.803                 1
## 10   2  16451390 242945309   21634  10469.350                 0
##    X8891.Log.R.Ratio X8892.Log.R.Ratio X8893.Log.R.Ratio
## 1                  0                 0                 0
## 3                  0                 0                 0
## 7                  0                 0                 0
## 8                  0                 0                 0
## 9                  0                 0                 0
## 10                 0                 0                 0
```

Take the case specific dataframe and subset the dataframe using only the probes and patient samples that are present in CGHcall object for consistency.

```
#Subset the case specific mBAFs
case.mBAFs     <- Threshold_mBAF_v3[[case]]

#Only use probes present in the CGHcall output
case.mBAFs     <- case.mBAFs[which(case.mBAFs$Name%in%featureNames(case_LogR_ratioCalled)),]

#Use only samples that are in CGHcall output
sample.cghcall <- which(unlist(strsplit(colnames(case.mBAFs),".B.Allele.Freq")) %in% unlist(strsplit(sampleNames(case_LogR_ratioCalled),".Log.R.Ratio")))
case.mBAFs     <- case.mBAFs[,c(1:3,sample.cghcall)]
head(case.mBAFs)
```

```
##          Name Chr Position X8890.B.Allele.Freq X8891.B.Allele.Freq
## 5   rs2980314   1   781258                  NA                  NA
## 6   rs6684487   1   791853                  NA                  NA
## 7   rs4245756   1   799463                  NA                  NA
## 8  rs12086311   1   808769                  NA                  NA
## 9  rs13303077   1   816617                  NA                  NA
## 10 rs28625089   1   840327           0.5431846           0.5505144
##    X8892.B.Allele.Freq X8893.B.Allele.Freq
## 5                   NA                  NA
## 6                   NA                  NA
## 7                   NA                  NA
## 8                   NA                  NA
## 9                   NA                  NA
## 10           0.5407525           0.5388075
```

The detection of CNN LOH is performed in a ‘for loop’ per chromosome. Once SNPs in the ‘AB state’ have been calculated and bins called as being in a state of LOH. Bins are merged to form regions when they are in the same consequetive state and the boundaries of these regions are merged if they are within the *con.bins.merge* number of bins. As region boundaries may overlap, these overlaps are removed and are *p*-end aligned (left aligned).

```
total.LOH.genome   <- NULL

for(chr in 1:22) {
  
  #Subset the mBAF data specific to this case
  case.chr.mBAFs <- case.mBAFs[which(case.mBAFs$Chr==chr),]
  
  #Calculate the positions of the bins, shifting by 10 SNPs  
  starts <- seq(1, nrow(case.chr.mBAFs)-(window-1), by=10)
  ends   <- seq(window, nrow(case.chr.mBAFs), by=10)
  binned.mbafs <- cbind(starts, ends)
  
  #Count the number of SNPs in the 'AB state' per bin for each sample
  for(s in (1:n.samples)+3) {
    hetero.mbaf.count <- NULL
    for (i in 1:nrow(binned.mbafs)) {
      count <- length(which(case.chr.mBAFs[binned.mbafs[i,1]:binned.mbafs[i,2],s]<=AB.state.threshold))
      hetero.mbaf.count <- c(hetero.mbaf.count, count)
    }
    binned.mbafs <- cbind(binned.mbafs, hetero.mbaf.count)
  }
  
  #If counts are equal to or lower than the LOH threshold, call as LOH
  binned.mbafs.states <- as.data.frame(binned.mbafs)
  for (s in (1:n.samples)+2) {
    for (i in 1:nrow(binned.mbafs.states)) {
      if(as.numeric(binned.mbafs.states[i,s]) <= loh.threshold) {
        binned.mbafs.states[i,s] <- 1
      } else {binned.mbafs.states[i,s] <- 0}
    }
  }
  
  #Add a status to each bin based on the combination of samples with LOH
  binned.mbafs.states$total <- apply(binned.mbafs.states[,(1:n.samples)+2], 1, function(x) paste0(x, collapse=""))
  
  #Give the bin a new identifier if it has a different status from the previous bin
  binned.mbafs.states$states   <- cumsum(c(1, binned.mbafs.states$total[-1] != binned.mbafs.states$total[-length(binned.mbafs.states$total)]))
  
  #Format new dataframe
  test <- data.frame(rep(chr, times=nrow(binned.mbafs.states)), 
                     binned.mbafs.states$starts, 
                     binned.mbafs.states$ends,
                     binned.mbafs.states$total,
                     binned.mbafs.states$states)
  colnames(test) <- c("CHR", "START", "END", "TYPE", "conseq")
  
  #Generate genomic regions of LOH by taking the start and end of consecutive bins with the same status 
  total <- do.call(rbind, 
                   by(test, list(test$CHR, test$conseq), 
                      function(df)
                        if( NROW(df) >=1 & df$TYPE[1] != paste0(rep("0", n.samples), collapse="") ) {
                          cbind(df[1, c("CHR", "START")] , df[NROW(df), c("END", "TYPE")] ) 
                        } else {NULL} ))
  
  #Smooth region boundaries if they are within con.bins.merge of each other
  if(!is.null(total)) {
    
    #Define region status as the same if they are within con.bins.merge of each other
    total$conseq <- cumsum(c(1, (as.numeric(rownames(total))[-1] - as.numeric(rownames(total))[-length(as.numeric(rownames(total)))]) >= con.bins.merge))
    
    #Collapse regions which are within con.bins.merge of each other
    total <- do.call(rbind, 
                     by(total, list(total$CHR, total$conseq), 
                        function(df)
                          if( NROW(df) >=1 & df$TYPE[1] != paste0(rep("0", n.samples), collapse="") ) {
                            cbind(df[1, c("CHR", "START")] , df[NROW(df), c("END")], df[1,"TYPE"] ) 
                          } else {NULL} ))
    colnames(total) <- c("CHR", "START", "END", "TYPE")
    
    #If the ends of regions overlap with the starts of the following region, highlight them as overlapping
    overlaps <- total$END[-length(total$END)] > total$START[-1]
    overlaps <- c(overlaps, FALSE)
    total <- data.frame(total, overlaps)
    
    #Make ends of overlapping region, equal to the start of the following region minus 1 (therefore not overlapping).
    for (i in 1:nrow(total)) {
      if(total[i,"overlaps"]) {
        total[i,"END"] <- total[i+1,"START"]-1
      }
    }
    total$overlaps <- NULL
    
  } else {
    #If there is no LOH detected, simply call the whole chromosome a region
    total <- cbind(test[1, c("CHR", "START")] , test[NROW(test), c("END", "TYPE")] ) }
  
  #collate data
  total.LOH.genome <- rbind(total.LOH.genome, total)
  
  #This is specific to this Rmarkdown file for displaying calculated data
  if(chr==2) {
    plotting.binned.mbafs        <- binned.mbafs
    plotting.binned.mbafs.states <- binned.mbafs.states
    plotting.total               <- total
  }
}

rownames(total.LOH.genome) <- NULL
```

*binned.mbafs* contains the number of SNPs in the AB state per sliding window and *binned.mbafs.states* is the ‘call’ of loss-of-heterozygosity based on the threshold *loh.threshold*.

The subsequent plots represent the *binned.mbafs* in black and the *binned.mbafs.state* calls in red of the copy number neutral LOH event in *case*. The threshold for calling loss-of-heterozygosity is displayed as a dashed line.

After smoothing boundaries of unique states, results from all chromosomes are stored in *total.LOH.genome*.

```
head(total.LOH.genome[,-4])
```

```
##   CHR START   END
## 1   1  4901  5070
## 2   1  6441  6570
## 3   2  2031  2140
## 4   2 11101 16640
## 5   2 16641 19640
## 6   2 19641 24090
```

Considering noisy arrays and/or copy number gains can introduce multiple regions we filter these regions by a size limit on the sizes of LOH as true CNN LOH often generate large regions when not affected by noise.

```
#Remove regions with no LOH
total.LOH.genome        <- total.LOH.genome[which(total.LOH.genome$TYPE!=paste0(rep(0, times=n.samples), collapse="")),]

#Use only large regions defined by LOH.size.threshold
total.LOH.genome        <- total.LOH.genome[which(total.LOH.genome$END-total.LOH.genome$START >= LOH.size.threshold),]

#Generate a GRanges object
total.LOH.genome.grange <- GRanges(seqnames=total.LOH.genome$CHR, ranges=IRanges(start=total.LOH.genome$START, end=total.LOH.genome$END))
total.LOH.genome.grange
```

```
## GRanges object with 4 ranges and 0 metadata columns:
##       seqnames         ranges strand
##          <Rle>      <IRanges>  <Rle>
##   [1]        2 [11101, 16640]      *
##   [2]        2 [16641, 19640]      *
##   [3]        2 [19641, 24090]      *
##   [4]       11 [    1,  3860]      *
##   -------
##   seqinfo: 2 sequences from an unspecified genome; no seqlengths
```

At this point we wish to incorporate our LOH regions into our regions generated from the Log R ratio data. In order to compare the two we replace the base pair position on the chromsome with the SNP number, as in the LOH regions.

```
regions.snps.genome <- NULL

for (chr in chr.vector) {

#Subset CGHcall output by chromosome
cgh.call.chr  <- case_LogR_ratioCalled[which(chromosomes(case_LogR_ratioCalled)==chr)]

#Subset genomic regions by chromosome
regions.chr   <- regions_dataframe_SNPs_sxrm_snpflt_dnsflt[which(regions_dataframe_SNPs_sxrm_snpflt_dnsflt$Chr==chr),]

#Replace bp locations by SNP number
regions.starts <- which(bpstart(cgh.call.chr)%in%regions.chr$Start)
regions.ends <- which(bpstart(cgh.call.chr)%in%regions.chr$End)

#Generate a new regions object with SNP index positions
regions.snps.chr <- data.frame(regions.chr$Chr, regions.starts, regions.ends, regions.chr[,6:ncol(regions.chr)])
regions.snps.genome <- rbind(regions.snps.genome, regions.snps.chr)

}

#Format regions
colnames(regions.snps.genome)[1:3] <- c("Chr", "Start", "End")
rownames(regions.snps.genome) <- NULL
```

Convert to GRanges object and search for overlaps between the LOH regions and the regions generated previously and merge them using the *disjoin()* in the Genomic Ranges package.

```
#Convert LRR regions with SNP index into a GRanges object
regions.snps.genome.granges <- GRanges(seqnames=regions.snps.genome$Chr,
                                       IRanges(start=regions.snps.genome$Start,
                                               end=regions.snps.genome$End))

#Find the LOH regions that occur within previously generated regions
overlaps <- findOverlaps(total.LOH.genome.grange, regions.snps.genome.granges, type="within")

#Remove regions that are not within the previously generated regions
total.LOH.genome.in.regions <- total.LOH.genome.grange[queryHits(overlaps)]

#Split the regions to include the LOH region
if(length(total.LOH.genome.in.regions) > 0) {
  
  #Merge the two GRanges objects
  disjoin.granges <- c(total.LOH.genome.in.regions, regions.snps.genome.granges)
  disjoin.granges <- sort(disjoin.granges)

  #Use the disjoin function to apply the split
  merged.LOH.regions <- disjoin(disjoin.granges)
  
} else {merged.LOH.regions <- regions.snps.genome.granges}
```

```
## 
## Attaching package: 'XVector'
## 
## The following object is masked from 'package:plyr':
## 
##     compact
```

Convert back into dataframe.

```
#Generate new dataframe from new regions GRanges object
regions.snps.loh.genome <- data.frame(as.numeric(as.vector(seqnames(merged.LOH.regions))), 
                                      start(merged.LOH.regions), 
                                      end(merged.LOH.regions))
colnames(regions.snps.loh.genome) <- c("chr", "regions.starts", "regions.ends")
regions.snps.loh.genome <- regions.snps.loh.genome[with(regions.snps.loh.genome, 
                                                        order(chr, regions.starts)), ]
rownames(regions.snps.loh.genome) <- NULL
```

Regions this smaller than 100 SNPs are removed by merging to smooth boundaries that may have not matched perfectly when previously disjoining the LOH regions and the LRR regions. This is defined by the *region.merger* parameter.

```
regions.snps.loh.genome.tmp <- NULL

for (chr in chr.vector) {
  
  #Subset CGHcall output by chromosome
  cgh.call.chr  <- case_LogR_ratioCalled[which(chromosomes(case_LogR_ratioCalled)==chr)]
  
  #Subset genomic regions by chromosome
  regions.chr   <- regions_dataframe_SNPs_sxrm_snpflt_dnsflt[which(regions_dataframe_SNPs_sxrm_snpflt_dnsflt$Chr==chr),]
  
  #Replace bp locations by SNP number
  regions.starts <- which(bpstart(cgh.call.chr)%in%regions.chr$Start)
  regions.ends <- which(bpstart(cgh.call.chr)%in%regions.chr$End)
  
  #Subset regions for the chromsome
  regions.snps.loh.chr <- regions.snps.loh.genome[which(regions.snps.loh.genome$chr==chr),]
  
  regions.snps.loh.chr.tmp <- NULL
  
  for(i in 1:length(regions.starts)) {
    
    #We will apply the smoothing for each region from the LRR data, therefore we need to subset
    region.subset.s <- which(regions.snps.loh.chr$regions.starts==regions.starts[i])  
    region.subset.e <- which(regions.snps.loh.chr$regions.ends==regions.ends[i])
    
    #This may now be more than one region in the LOH regions, where originally there was just one
    regions.snps.loh.region <- regions.snps.loh.chr[region.subset.s:region.subset.e,]
    
    #Classify region as the same if it is not greater than the merger
    regions.snps.loh.region$merger <- cumsum(c(1, (regions.snps.loh.region[,3][-1] - regions.snps.loh.region[,3][-nrow(regions.snps.loh.region)]) >= region.merger))
    
    #Merge regions if the difference is less than the merger
    regions.snps.loh.region <- do.call(rbind, 
                                    by(regions.snps.loh.region, list(regions.snps.loh.region$chr, regions.snps.loh.region$merger), 
                                       function(df)
                                         if( NROW(df) >=1) {
                                           cbind(df[1, c("chr", "regions.starts")] , df[NROW(df), c("regions.ends", "merger")] ) 
                                         } else {NULL} ))
    
    #Store output
    regions.snps.loh.chr.tmp <- rbind(regions.snps.loh.chr.tmp, regions.snps.loh.region)
        
  }
  
  #Remove classifier
  regions.snps.loh.chr <- regions.snps.loh.chr.tmp[,-4]
  
  #Store output for genome
  regions.snps.loh.genome.tmp <- rbind(regions.snps.loh.genome.tmp, regions.snps.loh.chr)
    
}
  
#Format output
regions.snps.loh.genome <- regions.snps.loh.genome.tmp
rownames(regions.snps.loh.genome) <- NULL
```

Get copy number call for each of the new regions we have generated.

```
new.regions.calls.genome <- NULL

for (chr in chr.vector) {
  
  #Subset CGHcall output by chromosome
  cgh.call.chr  <- case_LogR_ratioCalled[which(chromosomes(case_LogR_ratioCalled)==chr)]
  
  #Subset genomic regions by chromosome
  regions.chr   <- regions_dataframe_SNPs_sxrm_snpflt_dnsflt[which(regions_dataframe_SNPs_sxrm_snpflt_dnsflt$Chr==chr),]
  
  #Replace bp locations by SNP number
  regions.starts <- which(bpstart(cgh.call.chr)%in%regions.chr$Start)
  regions.ends <- which(bpstart(cgh.call.chr)%in%regions.chr$End)
  
  #Create new dataframe for calls
  regions.snps.calls <- data.frame(chr, regions.starts, regions.ends, regions.chr[,6:ncol(regions.chr)])
  
  #Use only chromosomal region from the new regions set we have made thus far
  regions.snps.loh.chr <- regions.snps.loh.genome[which(regions.snps.loh.genome$chr==chr),]
  
  new.regions.calls.chr <- NULL
  
  for(i in 1:nrow(regions.snps.loh.chr)) {
    
    #These are the calls for the new regions
    calls.vector.number <- which(regions.snps.loh.chr[i,"regions.starts"] >= regions.snps.calls[,"regions.starts"] & regions.snps.loh.chr[i,"regions.ends"] <= regions.snps.calls[,"regions.ends"])
    
    #This a new dataframe for this new region
    new.regions.calls.row <- data.frame(chr, regions.snps.loh.chr[i,"regions.starts"], regions.snps.loh.chr[i,"regions.ends"], regions.snps.calls[calls.vector.number,4:ncol(regions.snps.calls)])
    
    #Store output
    new.regions.calls.chr <- rbind(new.regions.calls.chr, new.regions.calls.row)
    
  }
  
  #Format output
  colnames(new.regions.calls.chr)[2:3] <- c("Start", "End")
  new.regions.calls.genome <- rbind(new.regions.calls.genome, new.regions.calls.chr)
}

#Format output
rownames(new.regions.calls.genome) <- NULL
```

Convert SNP numbers to base pair positions to re-produce a matrix of regions in the original format.

```
case.new.regions.genome <- NULL

for (chr in chr.vector) {
  
  #Subset CGHcall output by chromosome
  case.calls.chr <- case_LogR_ratioCalled[which(chromosomes(case_LogR_ratioCalled)==chr)]
  
  #Subset new regions by chromosome
  case.new.regions.chr <- new.regions.calls.genome[which(new.regions.calls.genome$chr==chr),]
  
  #Subset bp positions of new region boundaries for each chromosome
  bp.position.starts <- bpstart(case.calls.chr[case.new.regions.chr$Start])
  bp.position.ends   <- bpstart(case.calls.chr[case.new.regions.chr$End])
  
  #Create new regions dataframe
  case.new.regions.chr <- cbind(chr, bp.position.starts, bp.position.ends, case.new.regions.chr[,4:ncol(case.new.regions.chr)])
  
  #Format output
  colnames(case.new.regions.chr)[1:3] <- c("Chr", "Start", "End")
  
  #Store output
  case.new.regions.genome <- rbind(case.new.regions.genome, case.new.regions.chr)
}
```

We now have generated a new matrix of copy number change regions including copy number neutral LOH. We can use this data to calculate major and minor allele copy number for phylogenetic analysis.

Therefore we use the new regions, calls and mBAF data to calculate major and minor allele copy numbers by using the following equations:

> round(T \* M) = A

> T - A = B

Where:

1. T = Total copy number of region
2. M = Mean mBAF of region
3. A = Major allele copy number
4. B = Minor allele copy number

The major and minor allele copy numbers are outputted as FASTA files that are compatible with MEDICC.

```
#Calculate mean mBAF per region
case_mean_mBAF_per_regions <- mean_mBAF_per_region(case.new.regions.genome, case.mBAFs, chr_num=max(chr.vector))
```

```
#Format mean mBAFs dataframe
colnames(case_mean_mBAF_per_regions) <- c("Chr", "Start", "End", sampleNames(case_LogR_ratioCalled))
mBAF_per_region_sub <- case_mean_mBAF_per_regions[,4:ncol(case_mean_mBAF_per_regions)]

#Calculate total copy number per region
copynumber <- case.new.regions.genome[,4:ncol(case.new.regions.genome)]+2

#Calculate major and minor allele copy numbers
Major_A <- round(copynumber*mBAF_per_region_sub)
Minor_A <- copynumber-Major_A

#Create chromosome vector
C <- case_mean_mBAF_per_regions[,"Chr"]

#Format major allele output for FASTA files
chrom_major <- cbind(C,Major_A)
PerChrom <- list()
length(PerChrom) <- max(C)
for(l in 1:max(C)){
  PerChrom[[l]] <- subset(chrom_major,C==l)
}
MajorAppChrom <- PerChrom

#Format minor allele output for FASTA files
chrom_minor <- cbind(C,Minor_A)
PerChrom         <- list()
length(PerChrom) <- max(C)
for(l in 1:max(C)){
  cnum          <- C[l]
  PerChrom[[l]] <- subset(chrom_minor,C==l)
}
MinorAppChrom <- PerChrom

head(cbind(case.new.regions.genome[,1:3], Major_A), n=10)
```

```
##    Chr     Start       End X8890.Log.R.Ratio X8891.Log.R.Ratio
## 1    1    781258 120512303                 1                 1
## 2    1 145388137 147828939                 1                 1
## 3    1 149815079 249162263                 1                 1
## 4    2    106409  15619986                 1                 1
## 5    2  15626327  16445097                 2                 1
## 6    2  16451390 101690336                 1                 1
## 7    2 101691001 166781158                 2                 1
## 8    2 166786351 198938442                 2                 1
## 9    2 198944947 242945309                 2                 1
## 10   3    114133 197811684                 1                 1
##    X8892.Log.R.Ratio X8893.Log.R.Ratio
## 1                  1                 1
## 2                  1                 1
## 3                  1                 1
## 4                  1                 1
## 5                  1                 1
## 6                  1                 1
## 7                  1                 1
## 8                  2                 2
## 9                  2                 2
## 10                 1                 1
```

```
head(cbind(case.new.regions.genome[,1:3], Minor_A), n=10)
```

```
##    Chr     Start       End X8890.Log.R.Ratio X8891.Log.R.Ratio
## 1    1    781258 120512303                 1                 1
## 2    1 145388137 147828939                 1                 1
## 3    1 149815079 249162263                 1                 1
## 4    2    106409  15619986                 1                 1
## 5    2  15626327  16445097                 1                 1
## 6    2  16451390 101690336                 1                 1
## 7    2 101691001 166781158                 0                 1
## 8    2 166786351 198938442                 0                 1
## 9    2 198944947 242945309                 0                 1
## 10   3    114133 197811684                 1                 1
##    X8892.Log.R.Ratio X8893.Log.R.Ratio
## 1                  1                 1
## 2                  1                 1
## 3                  1                 1
## 4                  1                 1
## 5                  1                 1
## 6                  1                 1
## 7                  1                 1
## 8                  0                 0
## 9                  0                 0
## 10                 1                 1
```
