# Supplementary material for: Intra-Tumor Genetic Heterogeneity in Wilms Tumor: Clonal Evolution and Clinical Implications
Source: eBioMedicine. 2016 May 27;9:120–9. doi: 10.1016/j.ebiom.2016.05.029 (PMC4972528; doi:10.1016/j.ebiom.2016.05.029)
Supplement: Supplementary file 4 — Supplementary material 3 [file mmc4.zip › Genomic_waviness_documentation.html]

Genomic waviness detection using normal samples and normalisation per array


# Genomic waviness detection using normal samples and normalisation per array

This Rmarkdown file documents the detection of genomic waviness caused by flucuating GC content across the chromosome in normal sample arrays and illustrates how it can be removed by variance minimisation.

Firstly, we load the packages needed for this investigation. *zoo* is a package that contains the *rollMeans()* function for calculating moving averages.

```
library(zoo)
library(cluster)
library(plyr)
```

Next we load the LRR data for all cases in this investigation.

```
load("~/Wilms_tumour_project/results/2014-06-09/LogR_Ratios_all_v2.Rdata")
```

Here we document all our normal kidney and blood samples that will act as our normal samples for detection of genomic waviness.

```
#A vector of all the normal samples taken, not all were used to perform arrays
norm_n_blood <- c("9126",
                  "8703",
                  "8875",
                  "9199",
                  "8787",
                  "8814",
                  "8858",
                  "8856",
                  "8873",
                  "8891",
                  "10627",
                  "10892",
                  "10695",
                  "10685",
                  "11004",
                  "10905",
                  "9585",
                  "9775",
                  "9907",
                  "9967",
                  "8897",
                  "6604")
```

Our next step is to begin our analysis on only the autosomes, to calculate genomic waviness is the absence of gender differences.

```
#Create output for LRR for autosomes only
LogR_Ratios_sxrm <- LogR_Ratios_all

for(case in 1:length(LogR_Ratios_all)) {
  #Subset case
  case.LRR <- LogR_Ratios_all[[case]]
  
  #Remove sex chromosomes
  case.LRR$Chr <- as.numeric(as.character(case.LRR$Chr))

  #Remove the NAs that come from the X and Y chromosomes
  case.LRR <- case.LRR[which(!is.na(case.LRR$Chr)),]

  #Order the dataframe by chromosome and then position
  case.LRR <- case.LRR[with(case.LRR, order(Chr, Position)), ]
  
  LogR_Ratios_sxrm[[case]] <- case.LRR
}
```

The following plots show the genomic waviness present in chromosome 1 of case 16.

Subset the normal array from the original LRR list.

```
#A function to match sample IDs to corresponding columns in the LRR dataframe
find_matching_columns <- function(logr_data, column_names) {
                                  if(is.null(logr_data)) {
                                    return(NULL)
                                  }else {
                                    colnames_target <- colnames(logr_data)[4:ncol(logr_data)]
                                    colnames_query  <- paste0("X",column_names,".Log.R.Ratio")
                                    out <- logr_data[,1:3]
                                    for (i in 1:length(colnames_target)) {
                                      if (colnames_target[i] %in% colnames_query) {
                                        c <- logr_data[,i+3]
                                        out <- data.frame(out, c)
                                        colnames(out)[ncol(out)] <- colnames(logr_data)[i+3]
                                      }
                                    }
                                  return(out)}
}

#Subset LRR data by normal arrays
LogR_Ratios_norms <- lapply(LogR_Ratios_sxrm, find_matching_columns, norm_n_blood)
```

Next we combine all the normal arrays into a single dataframe.

```
#Collect first three columns
total_normals_data_frame <- LogR_Ratios_norms[[1]][,1:3]

#Make a dataframe with normal arrays from all patients
for (i in 1:length(LogR_Ratios_norms)) {
  if(ncol(LogR_Ratios_norms[[i]])>3){
    total_normals_data_frame <- data.frame(total_normals_data_frame, 
                                           LogR_Ratios_norms[[i]][,4:ncol(LogR_Ratios_norms[[i]])])
    colnames(total_normals_data_frame)[(ncol(total_normals_data_frame)-(ncol(LogR_Ratios_norms[[i]])-4)):ncol(total_normals_data_frame)] <- colnames(LogR_Ratios_norms[[i]])[4:ncol(LogR_Ratios_norms[[i]])]
  }
}

#Order dataframe by chromosomes and then position
total_normals_data_frame$Chr <- as.numeric(as.character(total_normals_data_frame$Chr)) 
total_normals_data_frame <- total_normals_data_frame[which(!is.na(total_normals_data_frame$Chr)),]
total_normals_data_frame <- total_normals_data_frame[with(total_normals_data_frame, order(Chr, Position)), ]
```

At this point we calculate the average LRR signal for each SNP probe individually.

```
#Calculate mean LRR for each SNP prone
total_normals_data_frame_mean <- data.frame(total_normals_data_frame, rowMeans(total_normals_data_frame[,4:ncol(total_normals_data_frame)], na.rm=TRUE))
colnames(total_normals_data_frame_mean)[ncol(total_normals_data_frame_mean)] <- "mean"
```

Next we calculate the moving average for each chromosome using a window size of 10 SNP probes.

```
#Create list to store the moving averages for each chromosome
movav_avnorm_chr <- list()
length(movav_avnorm_chr) <- 22
names(movav_avnorm_chr) <- c(1:22)

for (chr in 1:22) {
  #Subset dataframe by chromosome
  total_normals_data_frame_mean_chr <- total_normals_data_frame_mean[total_normals_data_frame_mean$Chr==chr,]
  
  #Subset only SNPs with a real value for their mean
    total_normals_data_frame_mean_chr_edit <- total_normals_data_frame_mean_chr[which(!is.na(total_normals_data_frame_mean_chr$mean)),]
  
  #Record which SNPs produces NAs
    nas <- which(is.na(total_normals_data_frame_mean_chr$mean))
  
  #Calculate moving average of means using window of size 10 and filling using NAs
    movav <- rollmean(total_normals_data_frame_mean_chr_edit$mean, 
                    10, 
                    fill=NA)
  
  #If some SNPs were NA re-enter these NAs into the results
    if(length(nas) > 0) {
    for (i in 1:length(nas)) {
    movav <- c(movav[1:(nas[i]-1)], NA, movav[nas[i]:length(movav)])
    }
    }
  
  #Store chromosome specific output
    movav_avnorm_chr[[chr]] <- movav
}

#Create vector of values for all autosomes
movav_avnorm_genome <- as.vector(unlist(movav_avnorm_chr))

#Save output
save(movav_avnorm_genome, file="movav_avnorm_genome.Rdata")
```

The following plot maps the genomic waviness against the normal samples taken from case 1.

Finally we define our normalisation function which measures the population variance of all the LRR data for the array after it has been subtracted by the genomic waviness multipled by a weight. By default the weight argument is 3 which results in testing weights from -3 to 3 in 0.1 intervals. The weight that produces the minimum variance is used to produce the final normalised sample.

```
movav_normalise <- function(sample, control, weight.lim=3) {
  ##Normalises samples LogR values based on the moving average of all normals
  ##Both objects must be same length
  popvar <- function(x, na.rm = FALSE) {
    # Calculates population variance instead of sample variance (which is the
    # default of the var() function in R).
    #
    # Args:
    # x: a vector of the population data.
    # na.rm: a logical value indicating whether NA values should be stripped
    # before the computation proceeds.
    #
    # Returns:
    # The population variance.
    if(na.rm) {
      x <- x[!is.na(x)]
    } else if(any(is.na(x))) {
      return(NA)
    }
    mean((x-mean(x))^2)
  }
  
  #r records the variance
  r <- NULL
  
  #Generate a vector of weights to be tested
  weights <- seq(-weight.lim,weight.lim, by=0.1)
  
  #Generate loop counter
  n <- 0
  
  for (i in weights) {
    #Count loop
    n          <- n+1
    
    #Subtract genomic waviness from sample using the weight co-efficient
    norm       <- sample - (control*i)
    
    #Calculate variance for the weight
    r[n] <- popvar(norm, na.rm=TRUE)
  }
  
  #Collect results in a dataframe
  results <- data.frame(weights, r)
  
  #Record the coefficient which produces the minimum variance
  final_weight <- results[which(results$r==min(results$r)),1]
  
  #Reproduce the normalised data using the best fitting coefficient
  output <- sample - (control*final_weight)
  
  #Print result
  cat("final weight is:",final_weight,"\n")
  
  return(output)
}

#Create output for the normalised arrays
normalised.LRR <- NULL

#Perform the normalisation on the first array
for(array in 1:7) {
normalised.output <- movav_normalise(LogR_Ratios_sxrm[[16]][,array+3], movav_avnorm_genome)

normalised.LRR <- cbind(normalised.LRR, normalised.output)
}
```

The following plots show how the genomic waviness has been removed from case 16, chromosome 1.

Now that we have completed the detection of genomic waviness for the autosomes, we will calculate a gender specific genomic waviness for the X chromosome.

```
#Create a function for subsetting just the X chromosome
find_X_chromosome <- function(logr_list){
  out <- logr_list[which(logr_list$Chr=="X"),]
  return(out)
}

#Subset the X chromosome from the original full data
LogR_Ratios_chrX <- lapply(LogR_Ratios_all, find_X_chromosome)

#Subset just the normal samples from the X chromsome dataframe
LogR_Ratios_norm_chrX <- lapply(LogR_Ratios_chrX, find_matching_columns, norm_n_blood)
```

Here we generate a chromosome X specific dataframe for the normal samples

```
#Subset first three columns for generating a X chromosome only dataframe
total_normals_data_frame_X <- LogR_Ratios_norm_chrX[[1]][,1:3]

#Generate an X chromosome only normal dataframe
for (i in 1:length(LogR_Ratios_norm_chrX)) {
  if(ncol(LogR_Ratios_norm_chrX[[i]])>3){
    total_normals_data_frame_X <- data.frame(total_normals_data_frame_X, 
                                           LogR_Ratios_norm_chrX[[i]][,4:ncol(LogR_Ratios_norm_chrX[[i]])])
    colnames(total_normals_data_frame_X)[(ncol(total_normals_data_frame_X)-(ncol(LogR_Ratios_norm_chrX[[i]])-4)):ncol(total_normals_data_frame_X)] <- colnames(LogR_Ratios_norm_chrX[[i]])[4:ncol(LogR_Ratios_norm_chrX[[i]])]
  }
}
```

Here we identify the female and male samples using partitioning around the mediods clustering. The LRR values are sufficently distinct enough that this naive method of identification is effective.

Manually enter the gender information is also possible.

Once we have the gender specific X chromosome dataframes we calculate the mean LRR value for each SNP.

```
#Cluster the X chromosome LRR data, which effectively identifies females and males
clustering_matrix <- total_normals_data_frame_X[,4:ncol(total_normals_data_frame_X)]
sex_clustering <- pam(t(clustering_matrix), k=2)
females <- which(sex_clustering$clustering==1)
males  <- which(sex_clustering$clustering==2)

#Subset dataframe by sex
females_normals_data_frame <- total_normals_data_frame_X[,c(1:3, females+3)]
males_normals_data_frame  <- total_normals_data_frame_X[,c(1:3, males+3)]

#Calculate mean for each SNP probe for each gender
females_normals_data_frame_mean <- data.frame(females_normals_data_frame, rowMeans(females_normals_data_frame[,4:ncol(females_normals_data_frame)], na.rm=TRUE))
males_normals_data_frame_mean <- data.frame(males_normals_data_frame, rowMeans(males_normals_data_frame[,4:ncol(males_normals_data_frame)], na.rm=TRUE))
colnames(females_normals_data_frame_mean)[ncol(females_normals_data_frame_mean)] <- "mean"
colnames(males_normals_data_frame_mean)[ncol(males_normals_data_frame_mean)] <- "mean"
```

Here we calculate the moving average of the means for the female X chromosome.

```
#Use only the means which have a real number
females_normals_data_frame_mean_edit <- females_normals_data_frame_mean[which(!is.na(females_normals_data_frame_mean$mean)),]

#Record the NAs
nas <- which(is.na(females_normals_data_frame_mean$mean))

#Calculate the moving average of the means using a window of size 10
movav <- rollmean(females_normals_data_frame_mean_edit$mean, 
                  10, 
                  fill=NA)

#Reintroduce the NAs
if(length(nas) > 0) {
    for (i in 1:length(nas)) {
      movav <- c(movav[1:(nas[i]-1)], NA, movav[nas[i]:length(movav)])
    }
}

#Store the female specific X chromosome genomic waviness
female.chr.X.movav <- movav

#Save the output
save(female.chr.X.movav, file = "female.chr.X.movav.Rdata")
```

Here we calculate the moving average of the means for the male X chromosome.

```
#Use only the means which have a real number
males_normals_data_frame_mean_edit <- males_normals_data_frame_mean[which(!is.na(males_normals_data_frame_mean$mean)),]

#Record the NAs
nas <- which(is.na(males_normals_data_frame_mean$mean))

#Calculate the moving average of the means using a window of size 10
movav <- rollmean(males_normals_data_frame_mean_edit$mean, 
                  10, 
                  fill=NA)

#Reintroduce the NAs
if(length(nas) > 0) {
  for (i in 1:length(nas)) {
    movav <- c(movav[1:(nas[i]-1)], NA, movav[nas[i]:length(movav)])
  }
}

#Store the male specific X chromosome genomic waviness
male.chr.X.movav <- movav

#As most LRR values are negative, subtract by the median LRR to centre values on zero
male.chr.X.movav.med.zero <- male.chr.X.movav - median(male.chr.X.movav, na.rm=TRUE)

#Save the output
save(male.chr.X.movav.med.zero, file = "male.chr.X.movav.med.zero.Rdata")
```
